# Dynamics of 5R-Tg Base Flipping in DNA Duplexes Based on Simulations—Agreement with Experiments and Beyond

**DOI:** 10.1021/acs.jcim.1c01169

**Published:** 2022-01-07

**Authors:** Shu dong Wang, Leif A. Eriksson, Ru bo Zhang

**Affiliations:** † School of Chemistry and Chemical Engineering, Beijing Institute of Technology, South Street no 5, Zhongguancun, Haidian District, 100081 Beijing, China; ‡ Department of Chemistry and Molecular Biology, University of Gothenburg, Medicinaregatan 9c, 405 30 Göteborg, Sweden

## Abstract

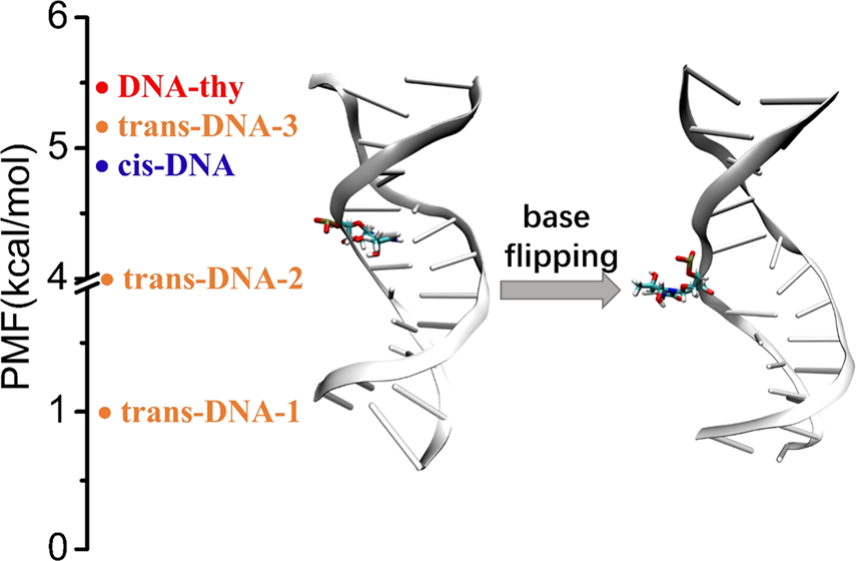

Damaged or mismatched DNA bases are normally thought to be able to flip out of the helical stack, providing enzymes with access to the faulty genetic information otherwise hidden inside the helix. Thymine glycol (Tg) is one of the most common products of nucleic acid damage. However, the static and dynamic structures of DNA duplexes affected by 5R-Tg epimers are still not clearly understood, including the ability of these to undergo spontaneous base flipping. Structural effects of the 5R-Tg epimers on the duplex DNA are herein studied using molecular dynamics together with reliable DFT based calculations. In comparison with the corresponding intact DNA, the *cis*-5R,6S-Tg epimer base causes little perturbation to the duplex DNA, and a barrier of 4.9 kcal mol^–1^ is obtained by meta-eABF for *cis*-5R,6S-Tg base flipping out of the duplex DNA, comparable to the 5.4 kcal mol^–1^ obtained for the corresponding thymine flipping in intact DNA. For the *trans*-5R,6R-Tg epimer, three stable local structures were identified, of which the most stable disrupts the Watson–Crick hydrogen-bonded G5/C20 base pair, leading to conformational distortion of the duplex. Interestingly, the relative barrier height of the 5R-Tg flipping is only 1.0 kcal mol^–1^ for one of these *trans*-5R,6R-Tg epimers. Water bridge interactions were identified to be essential for 5R-Tg flipping. The study clearly demonstrates the occurrence of partial *trans*-5R,6R-Tg epimer flipping in solution.

## Introduction

Base flipping is a key fundamental theme in nucleic acid biophysics and biochemistry. Studies have shown that base flipping is a common strategy for enzymes such as methyltransferases, glycosylases, and endonucleases,^1−6^ to read and chemically modify bases. Base flipping may even be linked to early events in the opening and unwinding of DNA for transcription and replication processes.^7^ Although extensive studies have found that many DNA repair/modification proteins completely flip their target base out extrahelically, it is still under debate whether the base flipping occurs spontaneously or not.^8−10^ Therefore, accurate information about base flip dynamics is of high interest and importance.^10,11^

Thymine glycol (5,6-dihydro-5,6-dihydroxy thymine; Tg) is the most common oxidation product of thymine. Approximately 400 Tg residues are formed in a normal cell each day, and 10–20% of genome damages have been attributed to the oxidative conversion of thymine to Tg.^12−18^ Due to the chirality of the C5 and C6 atoms, Tg could exist as a mixture of the two pairs of *cis*- and *trans*- stereoisomers: the 5R *cis–trans* pair (5R,6S: 5R,6R) and the 5S *cis–trans* pair (5S,6R: 5S,6S).^19−21^ The 5R-Tg stereoisomer is thought to be the more abundant of these, with an equilibrium ratio of 7:3 between *cis*-5R,6S and *trans*-5R,6R Tg in DNA oligomers containing the Tg·A base pair, while this ratio is 87:13% at the single-nucleoside level.^22,23^ The epimers are suggested to induce large structural changes to duplex DNA, reflected in the fact that the 5R-Tg base could be either extra-helical or coordinating to the opposing base on the complementary strand, depending strongly on the local interaction.^23,24^ The exact form or distribution would thus be linked to gene translation.^25−28^ Except for data from NMR spectra in solution in combination with 0.01 μs constrained molecular dynamics (rMD) simulations,^29^ reliable structural information related to Tg epimers is unfortunately not available. Although the Tg:adenine base pair is more biologically relevant, crystal structures are only available with cytosine opposite to 5R-Tg.^2,30^ It was previously thought that mismatched or damaged bases had a certain chance of spontaneously flipping out of the double helix structure of a DNA molecule because they could not form a normal and stable Watson–Crick base pair interaction^31^ and that the flipped base would thus function as a signal to be recognized and captured by repair proteins.^31−34^ This hypothesis lacks atomistic level evidence on the effect of particular Tg epimers on the DNA supramolecular structure in biologically relevant DNA.

**Scheme 1:**
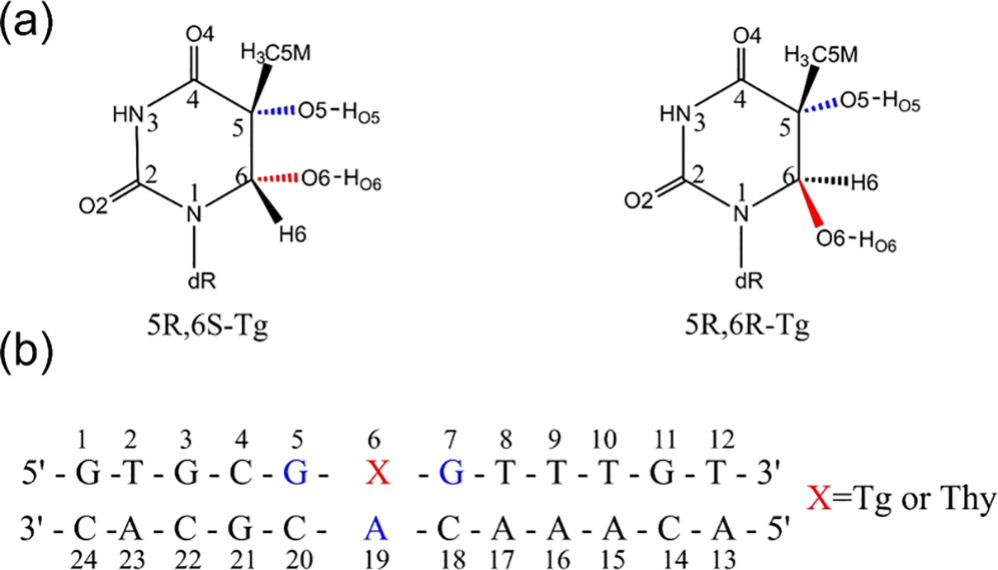
(a) The Structures of the 5R-Tg Pair and (b) the Sequence of the Dodecamer Used in the Current Study

In this work, the *cis*-5R,6S- and *trans*-5R,6R-Tg epimer-containing DNA duplexes, respectively, (referred to as *cis*-DNA and *trans*-DNA; see Scheme 1(29)) were modeled, and their static and dynamic structures and energies explored using Charmm36 force field based MD simulations. Benchmark calculations on the intact dodecamer with thymine in the same position (referred to as DNA-thy) were also performed to study how the 5R-Tg epimers deviate from the intact DNA duplex. The present results show that the *cis*-5R,6S-Tg in *cis*-DNA is always intrahelical and forms a Watson–Crick base pair with adenine. In contrast, three metastable conformations for *trans*-DNA are found. The locally most stable of these has relatively high energy and results in severe deformation of the duplex. This is attributed to the complex hydrogen-bonding network formed by *trans*-5R,6R-Tg with its surrounding bases, leading to loss of the classical Watson–Crick G/C and A/T pairs. The results also clearly illustrate the mechanism by which some of the *trans*-5R,6R-Tg conformers are capable of flipping out of the *trans*-DNA duplex.

## Computational Methods and Details

The initial coordinates of the *cis*-5R,6S-Tg containing dodecamer DNA (denoted *cis*-DNA) were obtained from the NMR structure in the Protein Data Bank (PDB ID: 2KH5),^29^ in which it is noted that the C5-CH_3_ group of Tg takes an axial position (Figure S1). Two additional dodecamer DNA duplexes were generated from the *cis*-5R,6S-Tg containing structure by mutating these in pymol,^35^ to form the systems containing *trans*-5R,6R-Tg (*trans*-DNA) and T (DNA-thy), the duplex sequence shown in Scheme 1b. DNA-thy is the intact DNA duplex, included as control.

Each dodecamer duplex was immersed in ca. 9260 TIP3P water molecules,^36^ in order to ensure that the systems were completely solvated. The system was neutralized by 0.15 M NaCl to imitate the intracellular environment. For nonbonded interactions, periodic boundary conditions with a cutoff radius of 12 Å were included, and the simulation box size was 59 × 63 × 44 Å with a minimum distance of 10 Å between DNA and the edges of the box. The particle mesh Ewald (PME) algorithm^37^ was used to handle electrostatic interactions. Bonds to hydrogen atoms were constrained using the SHAKE algorithm.^38^ The water molecules were initially minimized in 1000 conjugate gradient steps with the solute molecule(s) held fixed, followed by 1000 steps of conjugate gradient minimization of the whole system. After a 500 ps heating process from 0 to 298 K in a canonical ensemble (NVT) with the solute fixed, a series of harmonic constrained isothermal–isobaric ensemble (NPT) simulations were performed to enable a controlled release of the solute degrees of freedom. The scaling used for the constraints was 5.0, 1.0, and 0.5 kcal mol^–1^·Å^–2^, respectively. Under each constrained scaling, 500 ps MD simulation was carried out using an NPT ensemble. Constant temperature was maintained by the Langevin thermostat method^39^ and the pressure was maintained by the Langevin piston Nosé–Hoover method (a combination of the Nosé–Hoover constant pressure method^40^ and Langevin dynamics^39^). Unconstrained MD production simulations of 1 μs were performed in NPT ensembles with time step 2.0 fs. For *cis*-DNA, three independent 1 μs MD simulation were performed. For *trans*-DNA, an initial simulation of 1 μs length was first performed, which yielded the conformer *trans*-DNA-1. Two independent 1 μs replicas were subsequently performed with focus on the additional stable states, *trans*-DNA-2 and *trans*-DNA-3. In addition, the native DNA-thy (Scheme 1b) was simulated in three independent 1 μs MD simulations for comparison. The total simulation time in the study is more than 9.0 μs. The trajectory of the last 0.1 μs of each simulation was used to analyze and display the results using VMD 1.9.3.^41^ The DNA conformational analyses were performed with Curves+.^42^ All MD simulations were performed using NAMD 2.13^43^ together with the Colvar module.^44^ The Charmm36 general force field was used throughout.^45^

For insights into the flipping process of Tg from the duplex, we also performed the enhanced sampling dynamics, using the recently developed combination of extended adaptative biased force (eABF)^46,47^ and metadynamics (meta-eABF).^48^ In meta-eABF, a metadynamic-like memory kernel (MtD) is incorporated into the extended system alongside the eABF biasing force, thus, leading to
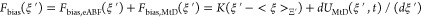
where *U*_MtD_(ξ′,*t*) is the time-dependent MtD-like memory kernel. The extended PMF then writes
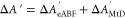


Through simultaneous addition of eABF biasing forces and a suitable form of the MtD Gaussian potentials, meta-eABF is particularly efficient for the rapid exploration of the free-energy landscape. The algorithm was proven to possess remarkable convergence properties over a broad range of applications including DNA, with as much as a 5-fold speedup, compared with standard ABF.^48,49^ The present average structures from the MD trajectories were used as initial structures for the potential of mean force (PMF) or free energy surface (FES) estimations. Meta-eABF was run under the NPT ensemble with instantaneous force values accrued in bins of width 0.1 × 0.1 Å. Settings for Gaussian hillWeight = 0.1 kcal mol^–1^ and hillWidth = 5 bin width were employed in the simulations. Distance is a reaction coordinate recently proposed to study base flipping.^50^ In this study, the center-of-mass (COM) separation distance between the Tg (or T6 in DNA-thy) nucleotide and A19 was considered as the collective variable (CV) in the meta-eABF simulation.

DFT calculations were performed at the isolated nucleotide level in a vacuum using the Gaussian 09 code,^51^ to assess the interaction of the non-Watson–Crick type of hydrogen-bonding base pairs. Geometry optimizations were performed using the M06-2X/6-31+G(d,p) method.^52^ The optimized structures were confirmed through frequency calculations at the same level, to be real minima with no imaginary vibration frequencies. The M06-2X functional was designed in part to yield more accurate noncovalent interactions containing significant dispersion contributions, as well as reliable thermochemical data.^53^ The interaction energy reported in the study is defined as Δ*E*_int_ = *E*_complex_ – (*E*_monomer1_ + *E*_monomer2_).

### Tg Parametrization

The partial atomic charges, bonds, angles, and dihedral terms were developed and fitted with the aid of the Force Field Toolkit (ffTK).^54^ while Lennard-Jones parameters and improper torsion parameters were taken by analogy from CHARMM’s CGenFF.^55^ All nonidentical atoms except hydrogens were assigned to unique atom types. We used the parametrization order as specified in the general CHARMM procedure, where the partial atomic charges were optimized first, followed by bonds and angles, and finally the dihedrals. The optimization of all parameters and vibrational analyses were done using the molecular geometries obtained by energy minimization at the MP2/6-31G* level of theory.

## Results and Discussion

### The *cis*-5R,6S Thymine Glycol Epimer in the DNA Dodecamer

**Figure 1:**
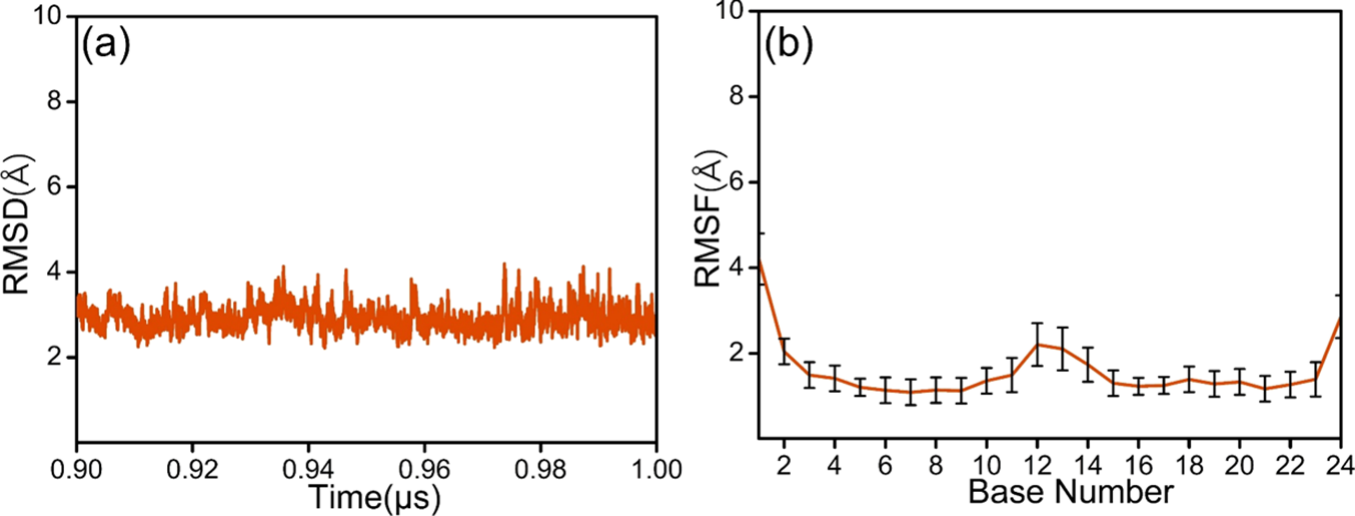
(a) RMSD (2.92 ± 0.29) and (b) nucleobase RMSF of the *cis*-DNA system during the last 0.1 μs of the MD simulation.

For the *cis*-DNA duplex, three independent 1 μs production simulations were performed and root-mean-square deviation (RMSD) with respect to the first frame of the production simulation was used to monitor the duplex structure as a measure of system stability, as displayed in Figure S2. The RMSD of *cis*-DNA displays only a slight fluctuation and yields very similar values to those of the intact duplex DNA-thy (Figure S3). Furthermore, the standard deviation in the RMSD over the last 0.1 μs simulation is only 0.40 Å (Figure 1a), showing that the relative structural change is very small. Therefore, in accordance with previous studies of natural and damaged DNA,^56^ detailed structural analysis was carried out on the last 0.1 μs simulation. In addition, root-mean-square fluctuation (RMSF) values were calculated to investigate how much the individual nucleobases moved during the simulations.^57^ As seen in Figure S2, the largest fluctuations occur at the terminal nucleotides of the duplex, again very similar to DNA-thy (Figure S3). The fluctuations of the *cis*-5R,6S-Tg base and its flanking G5 and G7 (cf. Scheme 1) are very small and positioned around RMSF values 1.17 ± 0.24, 1.74 ± 0.29, and 1.69 ± 0.29 Å, respectively. We may thus conclude that, in accordance with previous studies of DNA oligonucleotides,^56^ 1 μs simulations ensure convergence of key DNA structural parameters, and reliable conclusions can be drawn.

The conformation of the C5-CH_3_ group of thymine glycol has been thought to be a factor affecting the local structure of duplex DNA.^25,58,59^ Previous NMR experiments could not discriminate between axial or equatorial conformations of the CH_3_ group in *cis*-5R,6S-Tg, as both conformations showed agreement with the NOE data.^29,60^ Moreover, both conformations were observed in the 0.01 μs rMD simulation.^29^ The same results were obtained in the current study using rMD simulations with 0.15 kcal mol^–1^·Å^–2^ force toward the DNA duplex (Figure S4). Given the frequent change in conformation of the C5 methyl group in these rMD simulations, a more thorough investigation of the behavior of *cis*-5R,6S-Tg was called for. As a starting point in the extensive MD simulations, the CH_3_ group was placed in the axial position, based on the available NMR structure.

**Figure 2:**
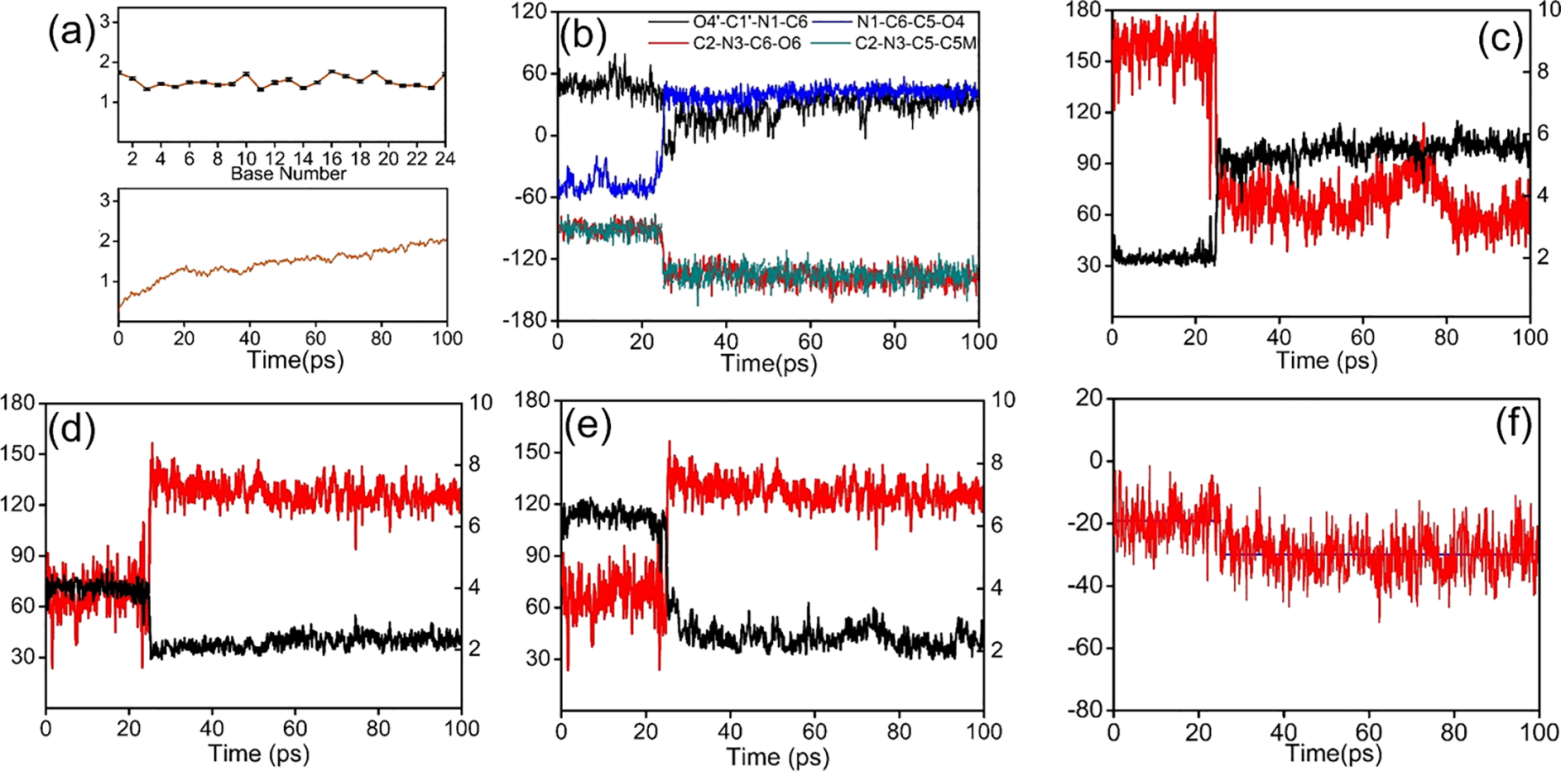
(a) RMSF (top, Å) and RMSD (bottom, Å) during the first 100 ps simulation of *cis*-DNA. (b) Relevant torsion angles (deg) of Tg. (c, d, e) Changes in hydrogen bonds Tg:O6H_O6_···N7:G7, Tg:O6H_O6_···O4′:Tg, and Tg:O6H_O6_···O5′:Tg, respectively. Length (Å), in black, right scale; angle (deg), in red, left scale. (f) Local energy (kcal mol^–1^) of the Tg + G7 dinucleotide.

**Figure 3:**
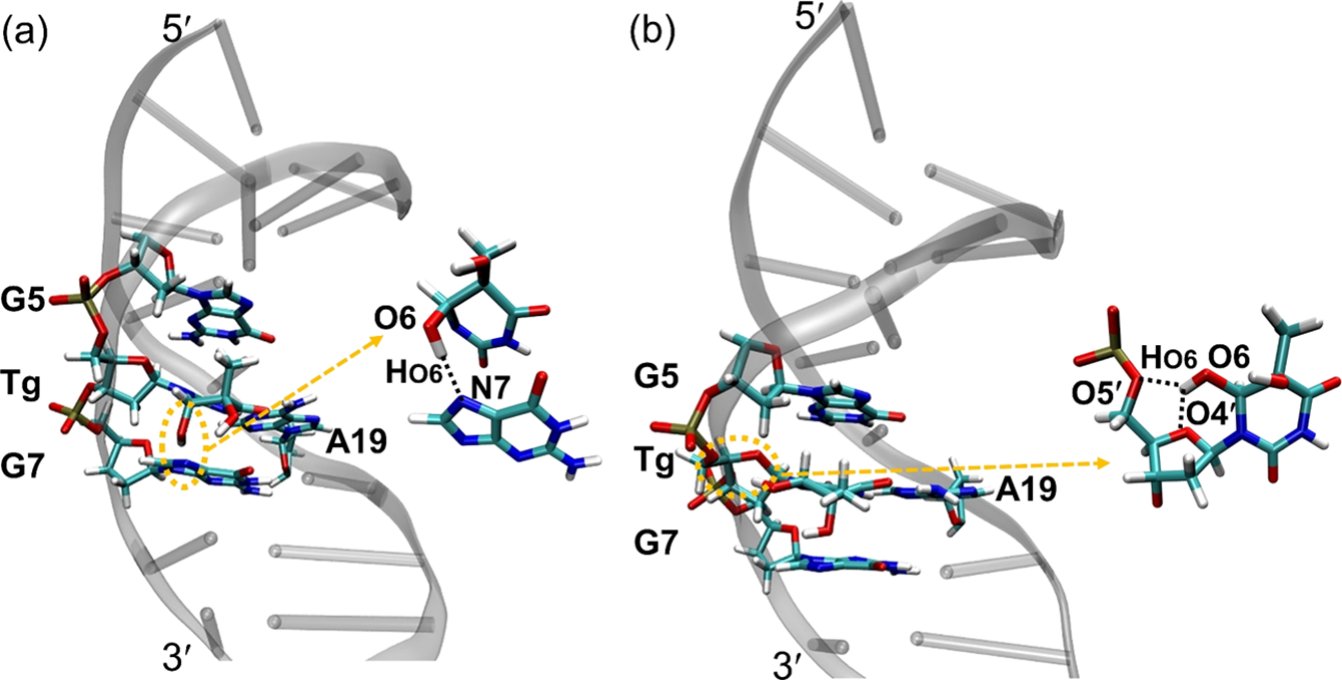
Average structures of (a) the first 25 ps simulation and (b) 25–100 ps simulation of *cis*-DNA. Relevant hydrogen bonds are indicated by dashed red lines. In a, Tg:O6H_O6_···N7:G7 = 2.03 Å; in b, Tg:O6H_O6_···O5′ = 2.36 Å; Tg:O6H_O6_···O4′ = 2.23 Å.

To explore the conformational selectivity of the CH_3_ group, the trajectories of the first 100 ps from 1 μs were analyzed separately; the RMSD and RMSF plots thereof are shown in Figure 2a. The conformational changes of the CH_3_ group and 6-OH group are described by the C2–N3–C5–C5M and C2–N3–C6–O6 torsion angles, respectively. During the first 25 ps, both the CH_3_ and 6-OH groups on *cis*-5R,6S-Tg were present in axial configuration with a dihedral angle of about 90°, as shown in Figure 2b and Figure 3a. The internucleotide interactions between *cis*-5R,6S-Tg and G7 are highlighted by the Tg:O6H_O6_···N7:G7 hydrogen bond, seen in Figure 3a. The hydrogen bond length is 2.03 ± 0.23 Å, and the relative occupancy^61^ of this hydrogen bond in the initial 25 ps of the trajectory is 96.8%. These are consistent with the observations made in the NMR experiments.^29^ After 25 ps, both the CH_3_ and 6-OH groups shift to the equatorial conformation, as seen in Figure 3b and Figure 2b, which is consistently maintained in all subsequent simulated trajectories. The *cis*-5R,6S-Tg:O6H_O6_···N7:G7 hydrogen bond disappears completely (Figure 2c). Instead, as shown in Figure 2d and e, Tg-intranucleotidyl O6H_O6_···O4′ and O6H_O6_···O5′ hydrogen bonds are formed with distances of 2.23 ± 0.20 and 2.36 ± 0.35 Å, respectively, with hydrogen-bond occupancies of 89.0% and 83.9% for the next 75 ps analyzed. The conformational changes were also studied by recording the energy of the *cis*-5R,6S-Tg and G7 containing dinucleotide during the simulation, seen in Figure 2f. We found average energies of approximately −19.1 and −30.0 kcal mol^–1^ for the two conformations, respectively, indicating that the equatorial configuration of the CH_3_ group in *cis*-5R,6S-Tg is thermodynamically preferred. One of the possible reasons is that the internucleotidyl *cis*-5R,6S-Tg:O6H_O6_···N7:G7 hydrogen bond is less stable than the two Tg intranucleotidyl O6H_O6_···O4′ and O6H_O6_···O5′ bonds. It thus appears that the conformational selectivity of the CH_3_ group on *cis*-5R,6S-Tg can be determined by local thermodynamics and controlled by the strength of the hydrogen bonds associated with the 6-OH substituent.

DFT was next used to explore the conformational selectivity. Structures of the *cis*-5R,6S-Tg nucleotide, including the methyl group in axial and equatorial conformations, were separately extracted from the MD simulations and optimized using the dispersion correction function M06-2X with the standard 6-31+G(d,p) basis set (Figure S5). The final single-point energy was computed at the MP2/6-311G(d,p) level (Table S1). For the system with axial conformation of the CH_3_ group, the 5-OH can form a hydrogen bond with either the O4 or O6 atom on *cis*-5R,6S-Tg, while 6-OH is unbound. These two rotamers are almost isoenergetic. They are significantly higher in energy (+3.5 kcal mol^–1^) than *cis*-5R,6S-Tg with an equatorial CH_3_ group, for which the two intranucleotidyl hydrogen bonds of 6-OH to O5′ and O4′ observed on the *cis*-5R,6S-Tg-nucleotide are the same as those observed in the MD trajectory. These results suggest that the CH_3_ group on *cis*-5R,6S-Tg either isolated or in double-stranded DNA will preferentially take an equatorial conformation. In addition, our MD simulations and DFT calculations show that the conformation of 6-OH in *cis*-5R,6S-Tg due to the ring puckering consistently displays the same conformation as the CH_3_ group.

The average structure of the stable *cis*-DNA dodecamer overlaps well with the intact DNA-thy structure (Figure S6a), except for the terminal nucleotides. The Watson–Crick type *cis*-5R,6S-Tg/A19 interaction and the two flanking G/C hydrogen bond pairs were always preserved and their stacking interactions well maintained throughout the simulation (Figures S7a and S8). The centroid–centroid distance between *cis*-5R,6S-Tg and G7 is 4.78 ± 0.25 Å, which exceeds the 3.90 ± 0.33 Å between T6 and G7 in DNA-thy (cf. Scheme 1 for numbering). This is due to the repulsive interaction between the axial 5-OH group and the G7 base, which is consistent with previous findings.^62^ The centroid distance between *cis*-5R,6S-Tg and G5 is 3.89 ± 0.15 Å.

**Figure 4:**
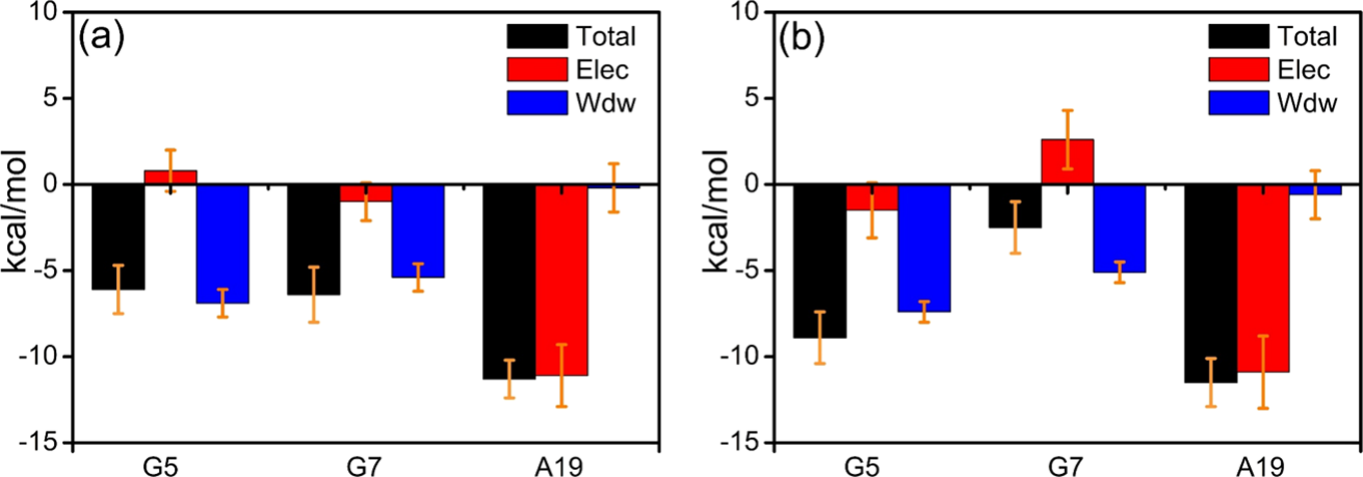
Interaction energy decomposition for (a) T and (b) 5R,6S-Tg with the adjacent G5, G7, and A19.

To further explore the effect of noncovalent interactions on the affinity, interaction energy decomposition analysis (EDA) was performed on the data from the MD simulations. The interaction energies of *cis*-5R,6S-Tg with its adjacent G5, G7, and A19 bases were decomposed during the last 0.1 μs of simulation, as shown in Figure 4b and Table S2. First, hydrogen bonding is readily identified as a critical interaction and is considered to be a key factor in maintaining the secondary structure of DNA. The Watson–Crick type T6/A19 hydrogen bond energy is approximately −11.2 ± 1.1 kcal mol^–1^, as obtained from the DNA-thy MD simulation (Figure 4a), in good agreement with the energy estimated at the M06-2X/6-31+G(d,p) level, −13.8 kcal mol^–1^. The average interaction energy between *cis*-5R,6S-Tg and A19 is −11.5 ± 1.4 kcal mol^–1^ (Figure 4b), which is very close to the −12.9 kcal mol^–1^ calculated at the M06-2X/6-31+G(d,p) level. These results indicate that the hydrogen bonding energies between A19 and T6 or *cis*-5R,6S-Tg are almost identical. In contrast, the electrostatic interaction, Elec, between G7 and *cis*-5R,6S-Tg is 2.6 ± 1.6 kcal mol^–1^ and the total interaction energy between the two is −2.5 ± 1.5 kcal mol^–1^. This is significantly weaker than that between *cis*-5R,6S-Tg and G5 and provides an explanation to why the distance between the *cis*-5R,6S-Tg and G7 bases is extended. The sum of the dominant van der Waals (vdW) interaction energy of *cis*-5R,6S-Tg with its flanking G5 and G7 bases is about −11.4 kcal mol^–1^, which is very close to the −11.5 kcal mol^–1^ obtained for the dominant Elec interaction energy of *cis*-5R,6S-Tg with A19. These indicate that Elec and vdW contribute equally to the affinity of *cis*-5R,6S-Tg to duplex DNA. The same is found in the interactions of T6 with A19, G5, and G7 in the intact DNA-thy. Moreover, the total interaction energies of T6 or *cis*-5R,6S-Tg with the A19, G5, and G7 bases are −23.8 and −22.9 kcal mol^–1^ in intact DNA-thy and *cis*-DNA, respectively. Thus, these comparative studies lead to the conclusion that the stability of the *cis*-5R,6S-Tg base in duplex DNA depends on the significant dispersion force of the *cis*-5R,6S-Tg nucleobase to its neighboring G5 and G7 bases, in addition to the hydrogen bond with the complementary A19 base. All replicas give highly similar results, seen in Table S3.

### The *trans*-5R,6R Thymine Glycol Epimer in the DNA Dodecamer

**Figure 5:**
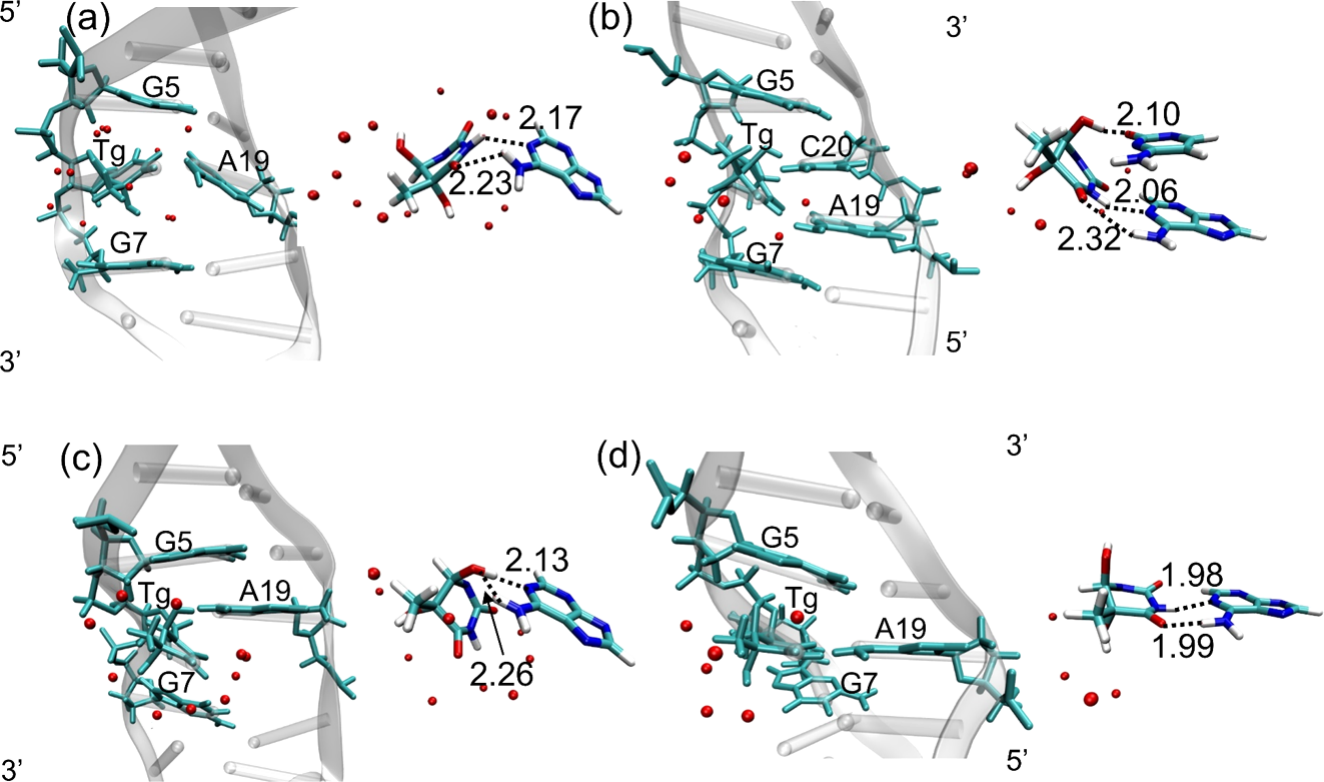
Average structures of (a) the metastable structure; (b) *trans*-DNA-1; (c) *trans*-DNA-2; (d) *trans*-DNA-3. Left: placement of the structures in the DNA duplex. Right: zooming in on the hydrogen bonded Tg-A19 base pairs. The red dots are oxygen atoms of water molecules, with hydrogen atoms omitted for clarity. Hydrogen bond lengths in Å.

**Figure 6:**
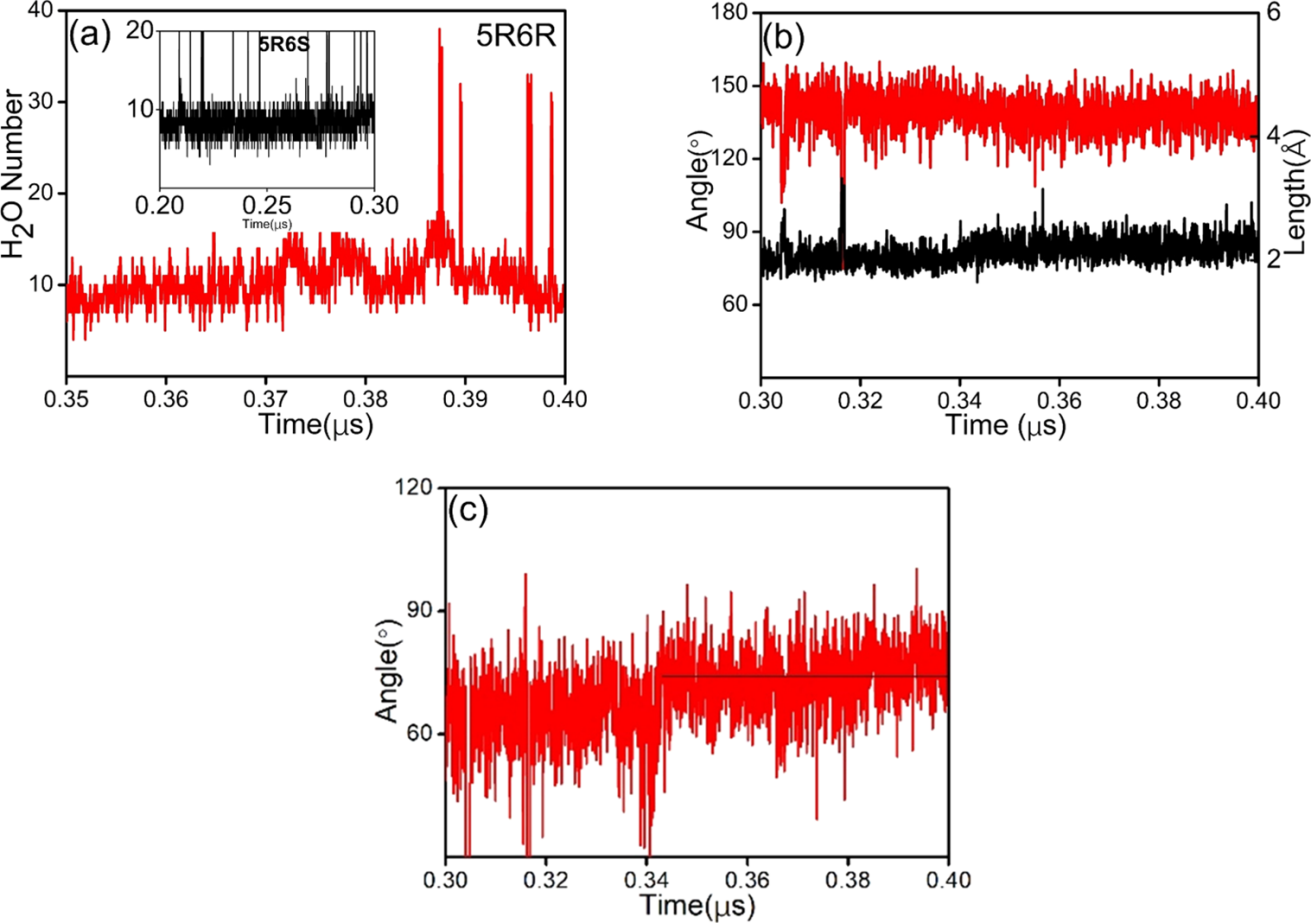
(a) Number of water molecules within 3.0 Å of *trans*-5R,6R-Tg in the metastable structure (in red) and *cis*-5R,6S-Tg (in black). (b) Change in Tg:O6H_O6_···O4′:Tg hydrogen bond length (Å, in black, right scale) and angle (deg, in red, left scale). (c) Torsion angle O4′–C1′–N1–C6 (deg).

5R-Tg was determined to be present in solution as a 7:3 equilibrium mixture of the *cis*-5R,6S- and *trans*-5R,6R-Tg epimers at 298 K.^22^ It is therefore difficult to discern experimentally how each epimer affects the structural and dynamic properties of the duplex DNA.^63,64^ On the basis of the current analysis of *cis*-5R,6S-Tg binding to the recognition site of the duplex DNA, we conclude that the bases G5, G7, and A19 surrounding *cis*-5R,6S-Tg play a key role in the binding and that *cis*-DNA essentially maintains the structure of the native system. To further investigate the effect of epimers on the stability of double-stranded DNA, a similar set of MD simulations and analyses was performed for the system containing the *trans*-5R,6R-Tg base. Throughout all trajectories, the CH_3_ group remained equatorial, and the 6-OH conformation axial. Interestingly, two relatively stable structures were identified in the 1 μs simulation. The first of these (referred to as the “metastable” state) was present in the simulation interval between 0.35 and 0.40 μs, and the RMSD and RMSF for all bases therein are shown in Figures S9a and S9b. An arc-shaped hydrogen-bonding between *trans*-5R,6R-Tg and A19 was noted, with an interaction energy estimated to be −8.6 ± 1.7 kcal mol^–1^ based on the MD trajectory. This indicates that their interaction strength was significantly lower than that of the hydrogen-bonded T6/A19 base pair in DNA-thy. Indeed, DFT calculations show that the isolated arched hydrogen-bonded base pair is not stable on the potential energy surface of the interaction between the *trans*-5R,6R-Tg and A19 bases. Instead, water molecules appear necessary to keep the metastable structure with the arc-shaped *trans*-5R,6R-Tg/A19 base pairing intact. As seen in Figure 5a and 6a, the number of water molecules around *trans*-5R,6R-Tg increases relative to that of 5R,6S-Tg in the *cis*-DNA production trajectories. The average water number around O6 and H_O6_ in *trans*-5R,6R-Tg is between 0.2 and 0.4 up until 0.35 μs of the simulation and increases to 1.1 and 1.2, respectively, in the metastable structure between 0.35 and 0.40 μs. In addition, around the *trans*-5R,6R-Tg nucleotide, the water number remains constant at 1.8 for O4′, 1.6 for O5, and 2.4 for H5 during the first 0.40 μs. The results indicate that the “additional” water molecule required to maintain the metastable structure causes a weakening of the hydrogen bond strength of Tg:O6H_O6_···O4′:Tg after 0.35 μs, presented in Figure 6b. It thereby becomes less capable of anchoring the *trans*-5R,6R-Tg base, which results in an increased rotation of the *trans*-5R,6R-Tg base around the N-glycosidic bond, Figure 6c. This is also consistent with the variation in RMSF values of 2.28 and 1.88 Å seen for the *trans*-5R,6R-Tg and A19 bases, respectively (Figure S9b).

**Figure 7:**
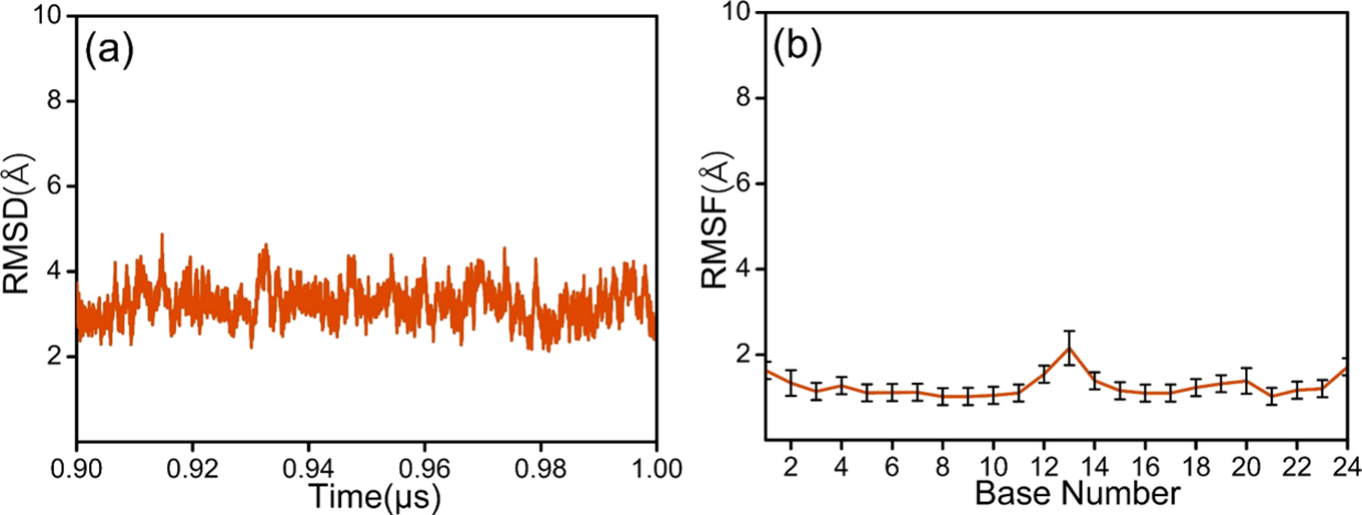
(a) RMSD (3.2 ± 0.41 Å) and (b) RMSF of *trans*-DNA-1.

**Figure 8:**
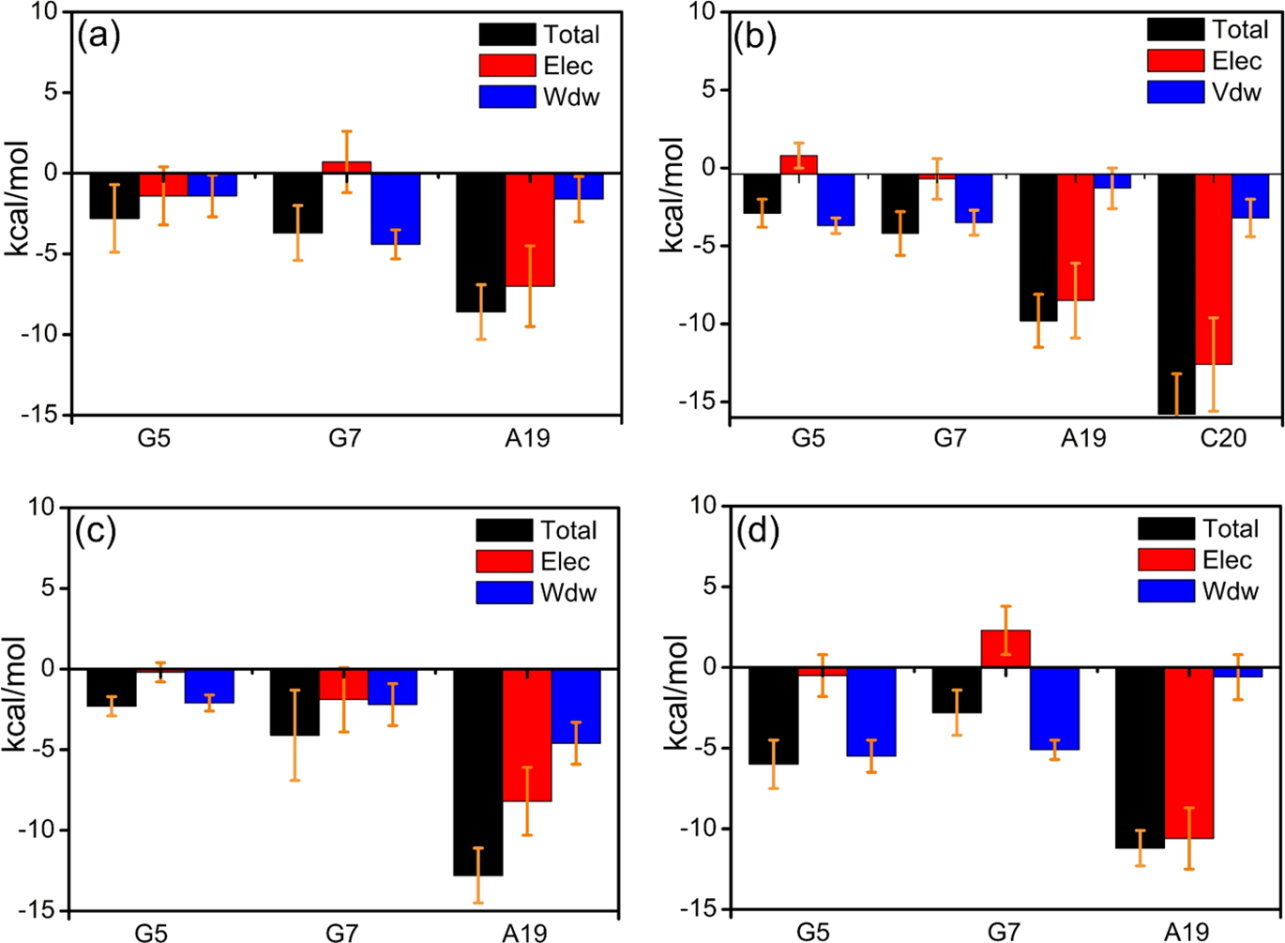
Interaction energy decomposition for *trans*-5R,6R-Tg interaction with the neighboring G5, G7, and A19 bases. (a) Metastable structure, (b) *trans*-DNA-1, (c) *trans*-DNA-2, (d) *trans*-DNA-3.

After the metastable structure, one of the observed stable *trans*-DNA species, *trans*-DNA-1, is formed at 0.40 μs and retained during the remaining simulation. The standard deviation in the RMSD (Figure 7a) over the last 0.10 μs is only 0.41 Å. Therefore, detailed structural analysis was thus carried out also on the last 0.1 μs simulation for this system. As shown in Figure 5b, the primary hydrogen bonding base pair G5/C20 is disrupted by the axial 6-OH of *trans*-5R,6R-Tg, resulting in the formation of a Tg:O6H_O6_···O2:C20 hydrogen bond with an average interaction energy of −15.8 ± 2.6 kcal mol^–1^, as shown in Figure 8b and Table S4. This result is in good agreement with the experimental proposal.^29,58^ The *trans*-5R,6R-Tg epimer causes a large distortion of the local structure of the duplex DNA in this conformation and is an explicit effect of base sequence in DNA. This is completely different from the duplex DNA with the *cis*-5R,6S-Tg epimer. Except for the Tg:O6H_O6_···O2:C20 hydrogen bond, the Watson–Crick hydrogen bond between *trans*-5R,6R-Tg and A19 is still retained with an average interaction energy of −9.8 ± 1.7 kcal mol^–1^. DFT calculations also confirmed the presence and stability of the *trans*-5R,6R-Tg interactions with A19/C20 (cf. Figure S10). The interaction energy is −25.6 ± 2.8 kcal mol^–1^, which is very close to the −24.6 kcal mol^–1^ obtained from the MD analysis. In addition to the phosphate and O4′ of the sugar ring being surrounded by water molecules, some water molecules also form hydrogen bonds with the O4 and O2 atoms of the *trans*-5R,6R-Tg base.

To further explore the *trans*-DNA structures, three additional simulation replicas of *trans*-DNA were performed. It should be noted that for all the *trans*-DNA replicas, after the initial stages with a planar *trans*-5R,6R-Tg/A19 base pairs, the metastable DNA structure is present with lifetimes from 0.02 to 0.05 μs. After the metastable state, *trans*-DNA is able to reach the more stable structures referred to as *trans*-DNA-1, -2, and -3 found in this study (see below).

The first of the replica simulations gave the same *trans*-DNA-1 structure as discussed above. In the other two replicas, two new stable structures of trans-DNA were also observed, and their RMSDs and RMSFs are shown in Figures S11 and S12. One of these, shown in Figure 5c, is labeled *trans*-DNA-2 and has a disrupted Watson–Crick hydrogen bond between the *trans*-5R,6R-Tg and A19. Instead, a hydrogen bond in the *trans*-5R,6R-Tg:O6H_O6_···N1:A19 is formed with a distance of 2.13 ± 0.25 Å. There are two water molecules close to the O2 and O4 atoms of *trans*-5R,6R-Tg. A third stable structure, labeled DNA-*trans*-3, was also found and is shown in Figure 5d. This structure forms a planar Watson–Crick *trans*-5R,6R-Tg/A19 hydrogen-bonding base pair, and no water molecules are close to the *trans*-5R,6R-Tg base. The average hydrogen-bonding energies of *trans*-DNA-2 and -3 are about −11.2 to −12.8 kcal mol^–1^, cf. Figure 8c,d and Table S4. The corresponding DFT calculations gave values of −13.4 and −13.7 kcal mol^–1^, respectively. Further structural analysis showed that the *trans*-5R,6R-Tg epimers in *trans*-DNA-2 and -3 barely perturb the structures of the adjacent base pairs.

Superposition of the observed structures show that *trans*-DNA-3 overlaps very well with DNA-thy (Figure S6d and Figure 5d), while the conformation of *trans*-DNA-1 deviates to a large extent from the intact DNA duplex (DNA-thy; Figure S6b). The distortions for *trans*-DNA-1 is mainly manifested by the change of the stable C20/G5 Watson–Crick base pair to a new hydrogen bonded structure between *trans*-5R,6R-Tg and C20 and A19, seen in Figure 5b. This also results in further reduction of the hydrogen-bond strength between *trans*-5R,6R-Tg and A19. A similar situation is found for the *trans*-DNA-2 structure, presented in Figure 5c. These results suggest that the *trans*-DNA-1 and -2 assemblies should be less stable than *trans*-DNA-3 with its perfect hydrogen-bonded base pairs. The stabilities of the *trans*-DNA species were roughly estimated by calculating the total energy of the G5/C20, Tg/A19, and G7/C18 pairs. Their relative stabilization energies are 0.0, −6.4, and −29.3 kcal mol^–1^, corresponding separately to the pairs in *trans*-DNA-1, -2, and -3. The stability order is consistent with the degree of deformation of *trans*-DNA.

### Flipping Free Energy Calculations

Understanding the dynamic process of the thymine glycol epimer affinity to the duplex DNA can provide further insights into the recognition mechanism of the modified nucleic acid by repair enzymes and polymerases.^2,28,30,65^ To address this issue, free-energy profiles were separately computed for 5R-Tg flipping out of the *cis*-DNA and *trans*-DNA supramolecular assemblies using a progressive sampling algorithm, meta-eABF.^46,48^ The same set of calculations was also performed for T6 flipping in DNA-thy, for comparison. In short, the center-of-mass (COM) separation distance between the Tg (or T6 in DNA-thy) nucleotide and A19 was considered as the collective variable (CV).

Previous studies of base flipping by MacKerell et al.,^66^ Lavery et al.,^67^ and us^68^ have shown that 200–220 ps simulation time for each umbrella sampling window is long enough for satisfactory convergence. Herein, we also examined the free energy surface (FES) of the 5R-Tg flipping using meta-ABF simulations of lengths 30, 40, 50, 60, 100, and 120 ns, respectively, for the *cis*-DNA system (Figure S13). For simulation times less than 40 ns, the free energy surfaces did not converge properly. For the trajectory time of 50 ns, the peak relative to a COM separation of 12.4 Å shows a free energy barrier of 4.7 kcal mol^–1^, which is slightly higher (by 1.7 kcal mol^–1^) than that obtained using 40 ns trajectories. The FES from the 60 ns simulation shows a free energy barrier of 4.9 kcal mol^–1^, which is very similar to that of the 50 ns simulation. In addition, comparative studies through prolonged simulation times (100 and 120 ns, respectively) show the estimated PMF to be 4.4–4.5 kcal mol^–1^, which is very close to the results obtained from the 60 ns simulation. In addition, the native thymine flipping FES from the 60 ns simulation shows a free energy barrier of 5.4 ± 0.2 kcal mol^–1^, comparable to the recent studies (5.3–7.5 kcal mol^–1^)^69−71^ and 7.1 kcal mol^–1^ from our meta-eABF simulation using pseudodihedral angle^72^ as the reaction coordinate (Figure S15). Our present results hence show that meta-eABF calculations require relatively short simulation times to meet satisfactory convergence.

**Figure 9:**
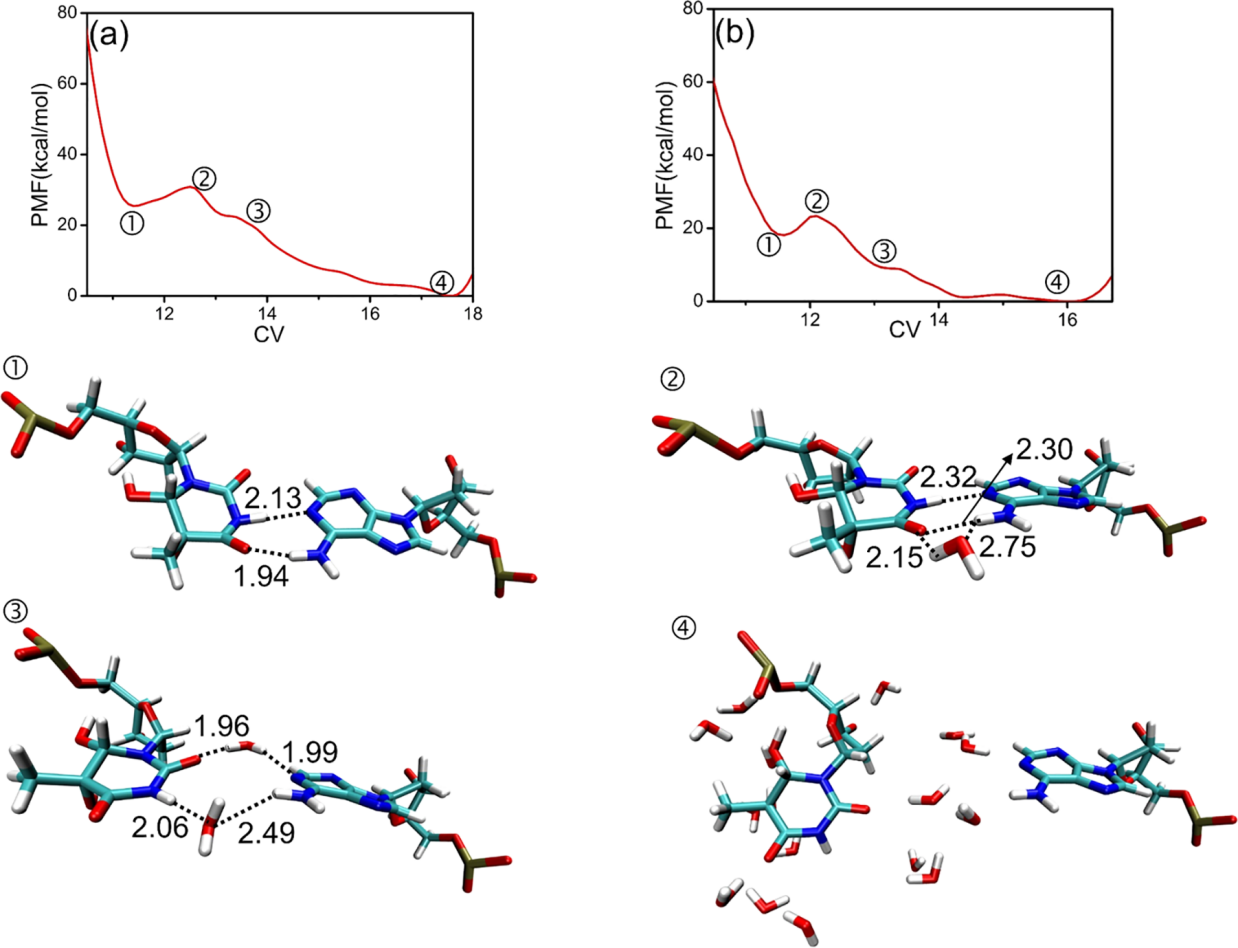
PMF profile of (a) *cis*-5R,6S-Tg (the error is within ca. 0.3 kcal mol^–1^) and (b) T (the error is within ca. 0.2 kcal mol^–1^) flipping out of the DNA duplex. Points 1–4 illustrate the main structural changes for *cis*-5R,6S-Tg and A19. The time evaluation plots of the examined CV in the meta-eABF simulations are shown in Figure S14. Hydrogen bond lengths in Å.

At the lowest point along the PMF curve (1 of Figure 9a), *cis*-5R,6S-Tg is bound by the hydrogen bonds and base-stacking interactions, and no water molecule is observed in the recognition region. From this first basin along the PMF curve, an energy barrier of 4.9 kcal mol^–1^ (2 of Figure 9a) must be overcome to break the hydrogen bond between A19 and *cis*-5R,6S-Tg. This is comparable to the 5.4 kcal mol^–1^ barrier obtained from the 60 ns simulation trajectory of T6 base flipping from DNA-thy (2 of Figure 9b). At this point, there is one water molecule forming a hydrogen-bonded bridge connecting A19 with *cis*-5R,6S-Tg, which further weakens the interaction between Tg and the DNA duplex. The base-stacking between Tg, G5, and G7 is retained during these initial stages of the process.

As the CV distance increases, a second water enters between *cis*-5R,6S-Tg and A19, disrupting the canonical Watson–Crick hydrogen bonds. After breaking the hydrogen bonds, a shallow basin (3 of Figure 9a) is found in the PMF. As an increasing number of water molecules penetrate into the active region, *cis*-5R,6S-Tg is flipped out from the helix and surrounded by water molecules, whereby the PMF curve reaches the lowest point (4 of Figure 9a). Apparently, water-mediated hydrogen bonding helps to reduce the activation barrier of the *cis*-5R,6S-Tg flipping. We conclude that the barrier height is comparable to that for T6 flipping out of the intact duplex DNA, implying that the *cis*-5R, 6S-Tg epimer should be very stable in the duplex DNA. Interestingly, the CH_3_ group is observed to shift between the axial and equatorial conformation during the 60 ns process, albeit with a preference for the equatorial arrangement as seen in Figure S16. Note that the crystal data showed that A19 was mutated into cytosine in the DNA-repair enzyme interaction system, causing *cis*-5R,6S-Tg to lose the Watson–Crick hydrogen bonds.^2,30^ Such a loss of base pairing would yield a much lower barrier toward flipping. In addition, the double helix structure of DNA deviates only a little from that of the standard B-DNA. The present results can provide a reasonable implication that the recognition of *cis*-5R,6S-Tg in DNA by repair enzymes yields a large deformation of the double strand to facilitate relevant repair of DNA.^2,30,73−75^

**Figure 10:**
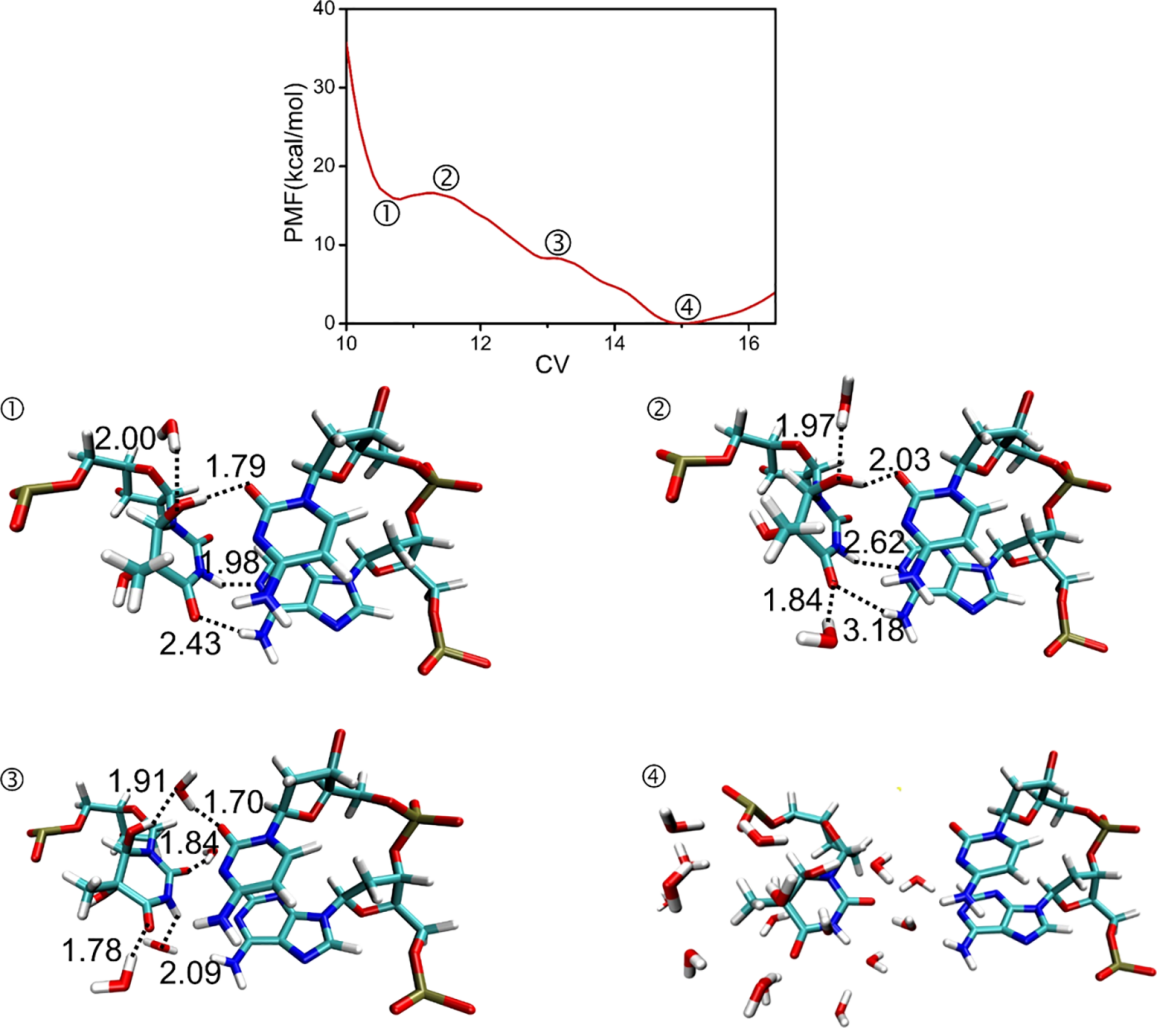
PMF profile of *trans*-5R,6R-Tg flipping out of the duplex of *trans*-DNA-1 (the error is within ca. 0.3 kcal mol^–1^). 1–4 illustrate the main structural changes of *trans*-5R,6R-Tg interaction with A19 and C20. The time evaluation plots of the examined CV in the meta-eABF simulations are shown in Figure S14.

PMF calculations for the *trans*-5R,6R-Tg base flipping processes were also performed. For *trans*-DNA-1, the stable local structure of *trans*-5R,6R-Tg/C20/A19 is solvated in the starting basin 1 (Figure 10) whereby one water molecule becomes bonded to 6-OH of Tg. Along the CV, a barrier height of only 1.0 kcal mol^–1^ needs to be overcome to break the hydrogen bonds between *trans*-5R,6R-Tg and the C20 and A19 bases (Figure 10). At the first peak 2, a second water comes close to O4 of *trans*-5R,6R-Tg. The second basin 3 is very shallow, representing the loss of hydrogen bonding between *trans*-5R,6R-Tg and A19,C20. Thereafter, complete solvation of the *trans*-5R,6R-Tg base is reached, point 4 in Figure 10. The low barrier is in sharp contrast to the barrier heights of 4.0 and 5.2 kcal mol^–1^ that must be overcome for elongation of the hydrogen bonding networks in *trans*-DNA-2 and *trans*-DNA-3, respectively (Figure S17), which instead are comparable to those calculated for *cis*-DNA and DNA-thy.

DNA helices are flexible and can exist in multiple conformations in solution. For the presently studied *trans*-DNA, three stable DNA duplexes were observed. They are in all likelihood in thermodynamic equilibrium in solution, in a ratio depending on the Boltzmann distribution. Thus, the distribution of the more distorted *trans*-DNA-1 should be smaller than those of *trans*-DNA-2 and -3. Strikingly, the barrier height for *trans*-5R,6R-Tg base flipping in *trans*-DNA-1 is significantly lower than for trans-DNA-2 and -3, implying that *trans*-5R,6R-Tg flips out of duplex DNA very easily in *trans*-DNA-1, once formed. This provides an explanation to the solution NMR experiments in which it was observed that the 5R-Tg bases in DNA were only partially extrahelical. By unambiguously taking the role of the *cis*–*trans* epimers into account, our study furthermore claims that the extrahelical 5R-Tg base should originate from the *trans*-5R,6R-Tg epimer in *trans*-DNA.

The DNA structural parameters of the obtained conformations were analyzed using Curves+.^42^ As seen from Table S5, the intrabase parameter buckle, opening and interbase parameter tilt of Tg·A base pair of *trans*-DNA-1 are much higher than those in DNA-thy and *cis*-DNA. These lead to significant distortions of the grooves near the Tg site. In particular, the major groove width of *trans*-DNA-1 increased to 18.6 Å compared to the major groove width of DNA-thy at ∼11.6 Å. Since a wider major groove was proven more favorable for base flipping,^76^ this explains why only a small barrier is required for *trans*-5R,6R-Tg flipping in *trans*-DNA-1.

**Figure 11:**
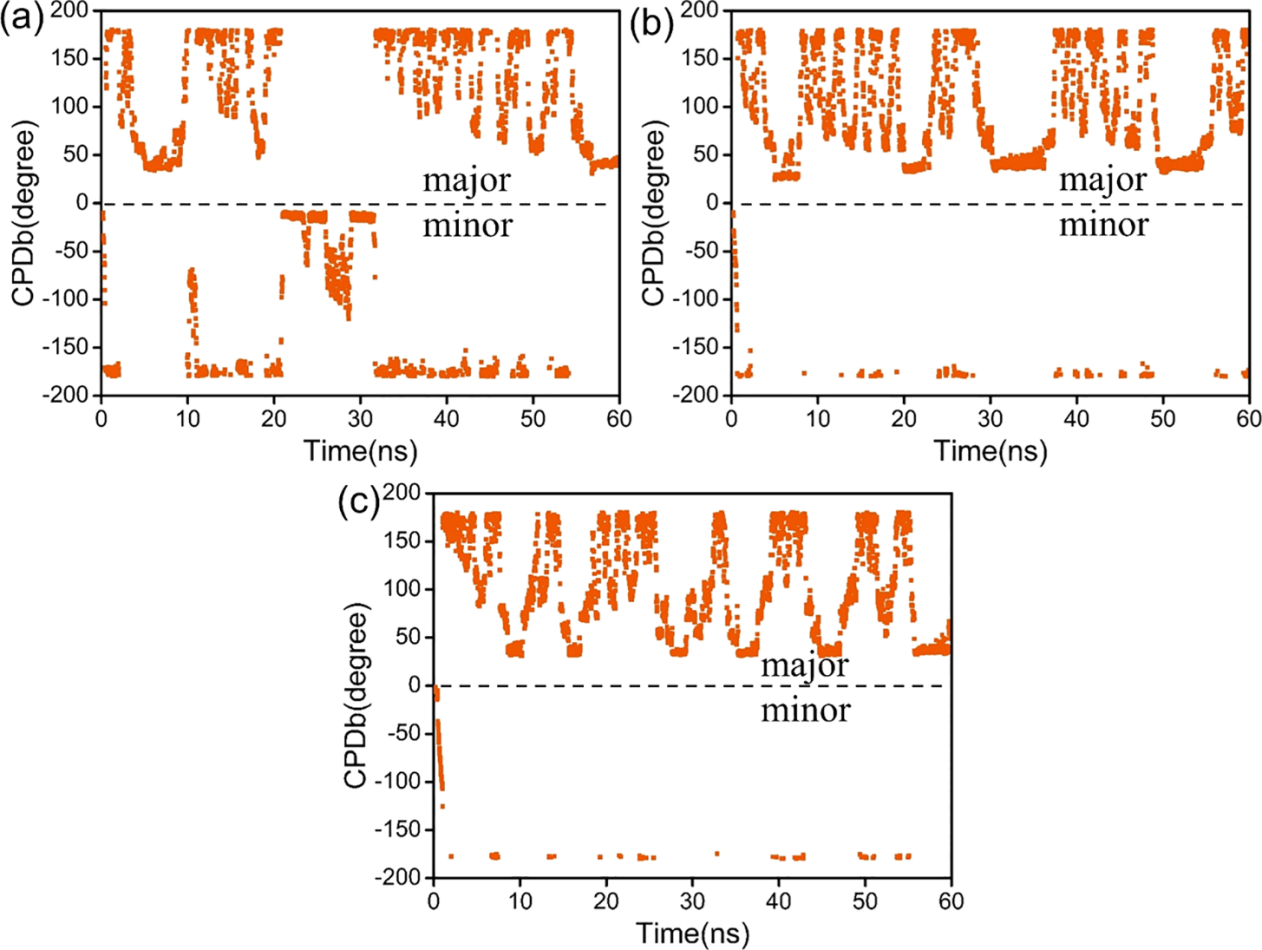
CPDb dihedral angle distribution of the Tg/T flipping out of the duplex during the simulation. (a) Native T in DNA-Thy, (b) *cis*-5R,6S-Tg in *cis*-DNA, (c) *trans*-5R,6R-Tg in *trans*-DNA-1. The CPDb dihedral angle is positive if T/Tg crosses into the major groove and negative if T/Tg crosses into the minor groove.

We adopted the pseudodihedral angle CPDb^72^ to study the pathway of Tg flipping, as shown in Figure 11. Native T (Figure 11a) can flip through both the major and the minor groove pathways, but the observed events for flipping through the minor groove pathway are fewer than through the major groove pathway. From Figures 11b,c and S18, we note that the base flippings of both *cis*-5R,6S-Tg and *trans*-5R,6R-Tg occur almost exclusively through the major groove pathway. A relatively larger steric barrier is found on the minor groove side, and thus the Tg base flipping in this direction seems to be largely forbidden. The present results are very consistent with previous studies.^77^

## Conclusions

Using the well-known thymine glycol as an example, we have used extended molecular dynamics simulations combined with reliable DFT calculations to address the influence of epimers on the stability of DNA supramolecular assemblies. This is to our knowledge the first comparative modeling study of DNA double-stranded structures including *cis*-5R,6S-Tg and *trans*-5R,6R-Tg epimers, respectively. It is clearly demonstrated that the CH_3_ group of 5R-Tg is energetically more inclined to be in a pseudoequatorial conformation due to the formation of stronger hydrogen bonds between 6-OH and O5′,O4′ in the 5R-Tg nucleotide.

The duplex DNA containing *cis*-5R,6S-Tg has comparable stability to the corresponding intact DNA. Energy decomposition analysis shows that Elec and vdW interaction contribute equally to the binding of *cis*-5R,6S-Tg to the duplex DNA. Three stable duplex structures containing *trans*-5R,6R-Tg were observed in our MD studies, depending on their surrounding bases and the influence of water. In the replicas of *trans*-DNA, an arched hydrogen-bonded *trans*-5R,6R-Tg/A19 pair is present as a metastable structure in the trajectories, preceding each of the stable species. The most stable local structure is unambiguously found in *trans*-DNA-1, indicating a complex hydrogen bonded network between *trans*-5R,6R-Tg and the A19 and C20 bases, which points to a clear base sequence effect. The stable local structure is also demonstrated using DFT calculations.

The activation barrier for 5R,6S-Tg flipping out of the duplex DNA in *cis*-DNA is ca. 4.9 kcal mol^–1^. This is comparable to the 5.4 kcal mol^–1^ computed for T6 base flipping in native DNA, showing that *cis*-5R,6S-Tg is stably positioned in the duplex DNA and will not easily attain an extrahelical position. However, the activation barrier for *trans*-5R,6R-Tg to flip out of the double helix DNA ranges from 1.0 to 5.2 kcal mol^–1^, depending on its local structure. Due to the conformational equilibrium of the flexible *trans*-DNA species in solution, the population of *trans*-DNA-1 with the most stable local structure should be the smallest due to loss of the classical Watson–Crick hydrogen-bonded base pair structure. Moreover, *trans*-DNA-1 displayed the smallest barrier height for *trans*-5R,6R-Tg flipping among the studied *trans*-DNA species in solution. The results provide detailed structural information on the 5R-Tg epimer in a DNA duplex and can serve as a basis for understanding the recognition of the 5R-Tg epimer by repair enzymes.

## Supporting Information Available

RMSD and RMSF data and various structural analyses from the MD simulations, optimized structures and energies, and PMF profiles from meta-eABF simulations (PDF)
