# Supplementary material for: Dynamics of 5R-Tg Base Flipping in DNA Duplexes Based on Simulations—Agreement with Experiments and Beyond
Source: J Chem Inf Model. 2022 Jan 7;62(2):386–98. doi: 10.1021/acs.jcim.1c01169 (PMC8790752; doi:10.1021/acs.jcim.1c01169)
Supplement: Supplementary file 1 — ci1c01169_si_001.pdf [file ci1c01169_si_001.pdf]

Supporting Information for

Dynamics of 5R-Tg Base Flipping in DNA Duplexes based on Simulations – Agreement with Experiments and Beyond

Wang Shu dong[1], Leif A. Eriksson[2*] and Zhang Ru bo[1*]

[1]School of Chemistry and Chemical Engineering, Beijing Institute of Technology, South Street no 5, Zhongguancun, Haidian District, 100081 Beijing, China, and [2]Department of Chemistry and Molecular Biology, University of Gothenburg, Medicinaregatan 9c, 405 30 Göteborg, Sweden.

Table of Contents

Figure S1. Axial or equatorial conformation of the 5-CH_3 group......3

Figure S2. RMSD and RMSF of the three independent 1 μs simulation of **cis-DNA......4**

Figure S3. RMSD and RMSF of the three independent 1 μs simulation of **DNA-thy......5**

Figure S4. The RMSD, torsion angle N1-C6-C5-C4 and C2-N3-C5-C5M and the **Tg**:O6H_{O6}•••N7:G7 hydrogen bond of the 0.01 μs rMD simulation......6

Figure S5. The optimized structures of **5R6S-Tg**......7

Table S1. The single-point energy of **5R6S-Tg** calculated at different level......7

Table S2. Interaction energy decomposition (kcal mol^{-1}) of T and **5R,6S-Tg** with its adjacent bases G5, G7 and A19......8

Table S3. Interaction energy decomposition (kcal mol^{-1}) of T and **5R,6S-Tg** with its adjacent bases G5, G7 and A19 for the replicas......8

Table S4. Interaction energy decomposition of **5R,6R-Tg** with the adjacent G5, G7 and A19/C20 ($kcalmol^{-1}$)...... 8

Figure S6. Overlap of average structures......9

Figure S7. The average structures of **cis-DNA** and **DNA-thy**......10

Figure S8. The distance of Tg with G5, G7 of the last 0.1 μs simulation......10

Figure S9. The RMSD and RMSF of **trans-DNA-1** 11

Figure S10. Optimized structures of **5R6R-Tg** with A19/C20 for **trans-DNA**......11

Figure S11. The RMSD and RMSF of **trans-DNA-2**......12

Figure S12. The RMSD and RMSF of **trans-DNA-3**......13

Figure S13. Free energy profiles as a function of the CV for different flip times and the corresponding time evaluation plot of the examined CV of the meta-eABF......14

Figure S14. Time evaluation plot of the examined CV of the meta-eABF simulations of **DNA-Thy**, **cis-DNA** and **trans-DNA-1**......14

Figure S15. PMF profiles of **T/Tg** flipping out of the duplex with pseudo-dihedral angle CPDb as the reaction coordinates..15
Figure S16. Change in 5R6S-Tg torsion angle C2-N3-C5-C5M during the 60ns pmf process...........16
Figure S17. PMF profiles of **5R6R-Tg** flipping out of the duplex for **trans-DNA-2** and **trans-DNA-3**..16
Figure S18. The CPDb dihedral angel distribution of the Tg flipping out of the duplex along the simulation time..17
Figure S19. Comparison of QM and MM potential energies of dihedral scans performed for **5R6S-Tg** and **5R6R-Tg** in order to optimize the dihedral parameters..17
Table S5 Helical, backbone and groove parameters calculated with Curves+...................................18

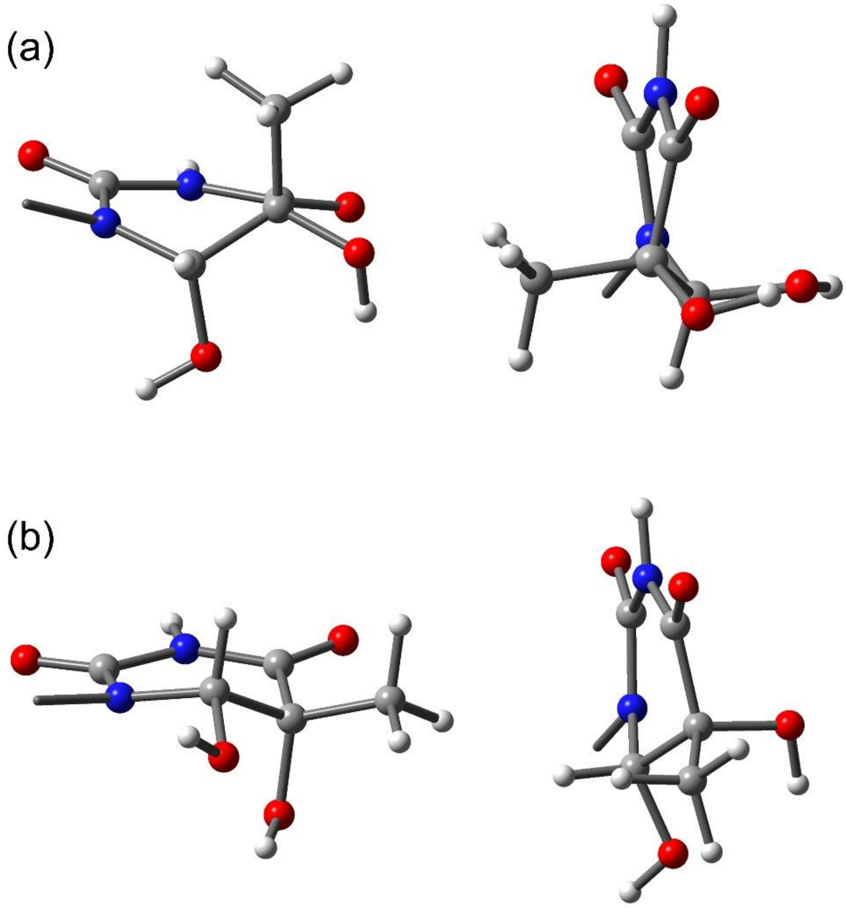

Fig. S1 (a) Axial or **(b)** equatorial conformation of the 5-CH_3 group. Axial or equatorial denote the pseudo axial or pseudo equatorial orientation of the 5-CH_3 group at C5. The conformational changes of the 5-CH_3 group is described by the C2-N3-C5-C5M dihedral angle. Red denotes oxygen and blue denotes nitrogen.

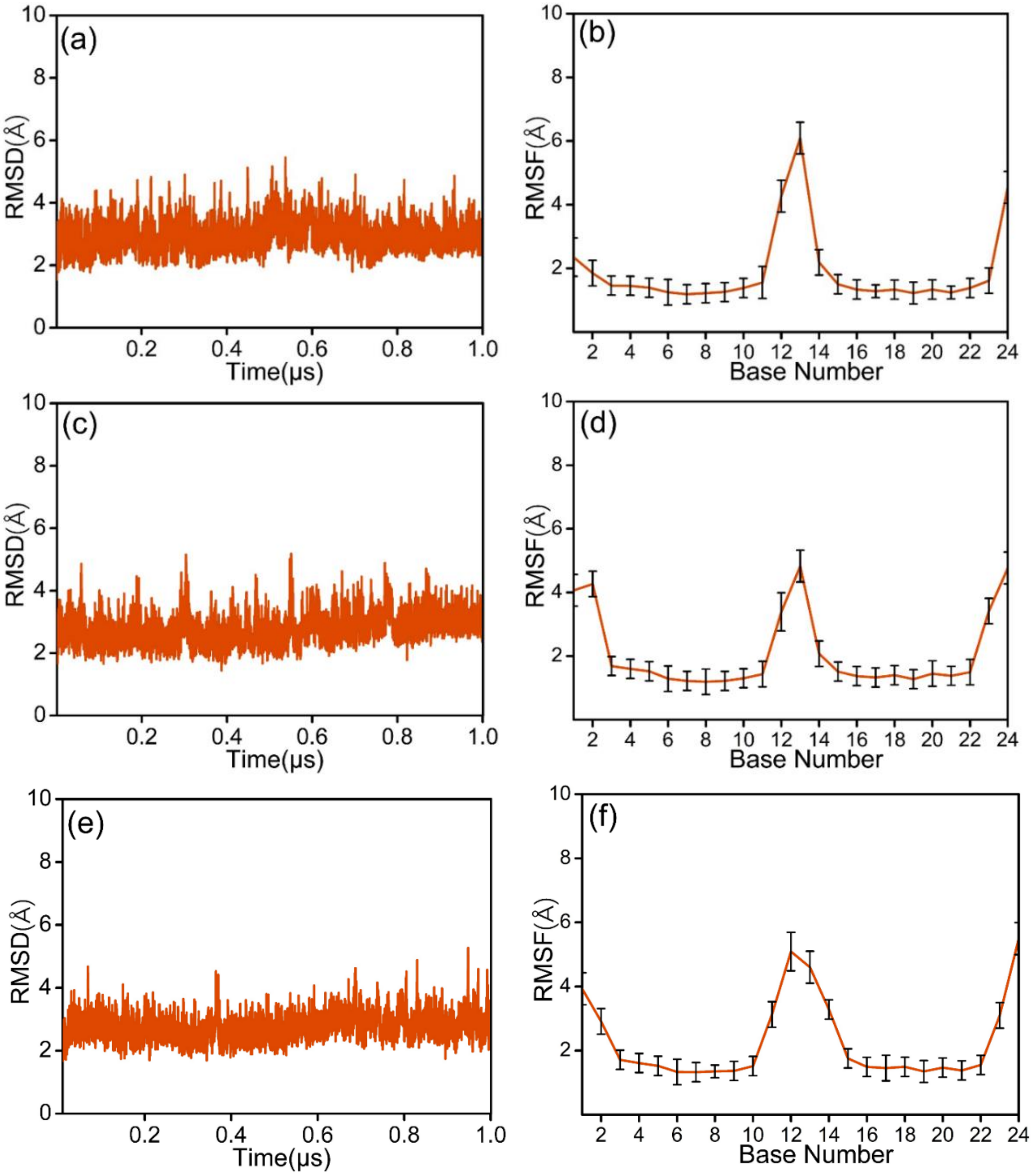

Fig. S2 (a) RMSD (2.93±0.43) and **(b)** RMSF of $1\mu s$ simulation for **cis-DNA** replica 1**; (c)** RMSD (2.75±0.47) and **(d)** RMSF of $1\mu s$ simulation for **cis-DNA** replica 2; **(e)** RMSD (2.73±0.39) and **(f)** RMSF of $1\mu s$ simulation for **cis-DNA** replica 3.

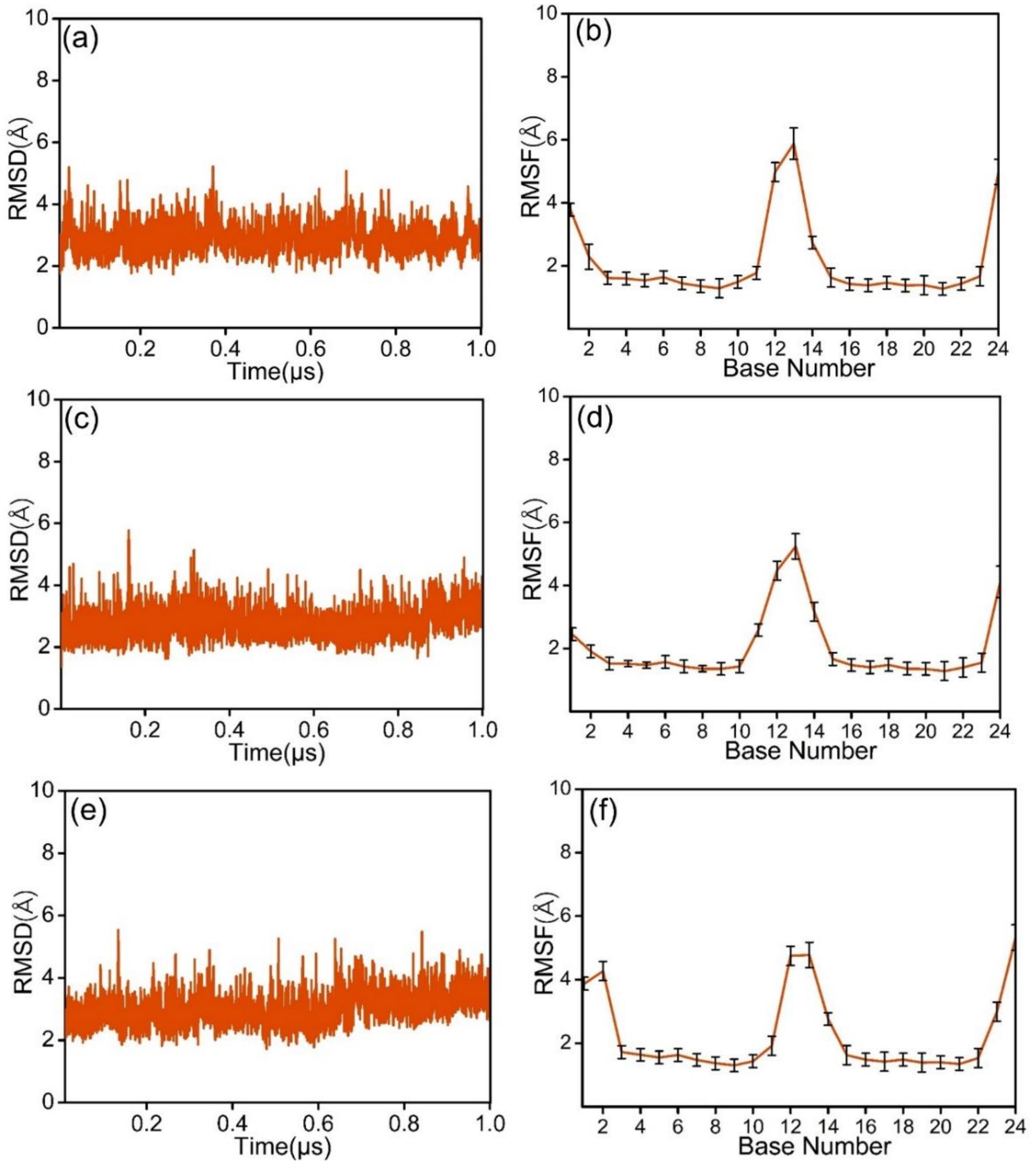

Fig. S3 (a) RMSD (2.91±0.45) and **(b)** RMSF of 1 μs simulation for **DNA-thy** replica 1**; (c)** RMSD (2.78±0.47) and **(d)** RMSF of 1 μs simulation for **DNA-thy** replica 2; **(e)** RMSD (3.01±0.48) and **(f)** RMSF of 1 μs simulation for **DNA-thy** replica 3.

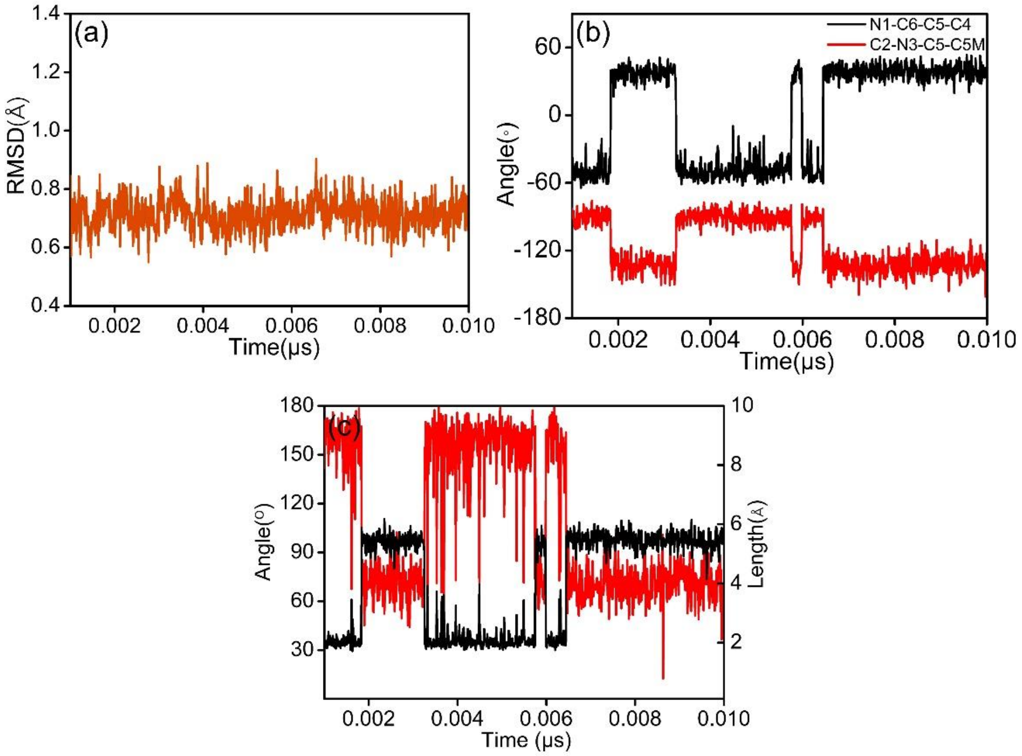

Fig. S4 Data from the 0.01 μs rMD simulation. **(a)** RMSD (0.71±0.05Å). **(b)**Torsion angles N1-C6-C5-C4 and C2-N3-C5-C5M. **(c)** Length (black) and angle (red) of the Tg:O6H_{O6}•••N7:G7 hydrogen bond. As see in the figures, at t=0.002 μs, the 5R-Tg CH_3 group shifts from an axial to an equatorial conformation for the first time, and shifts back to axial position at t=0.0035 μs. The 5R-Tg CH_3 group then shifts back to the equatorial conformation for a second time at 0.0065 μs.

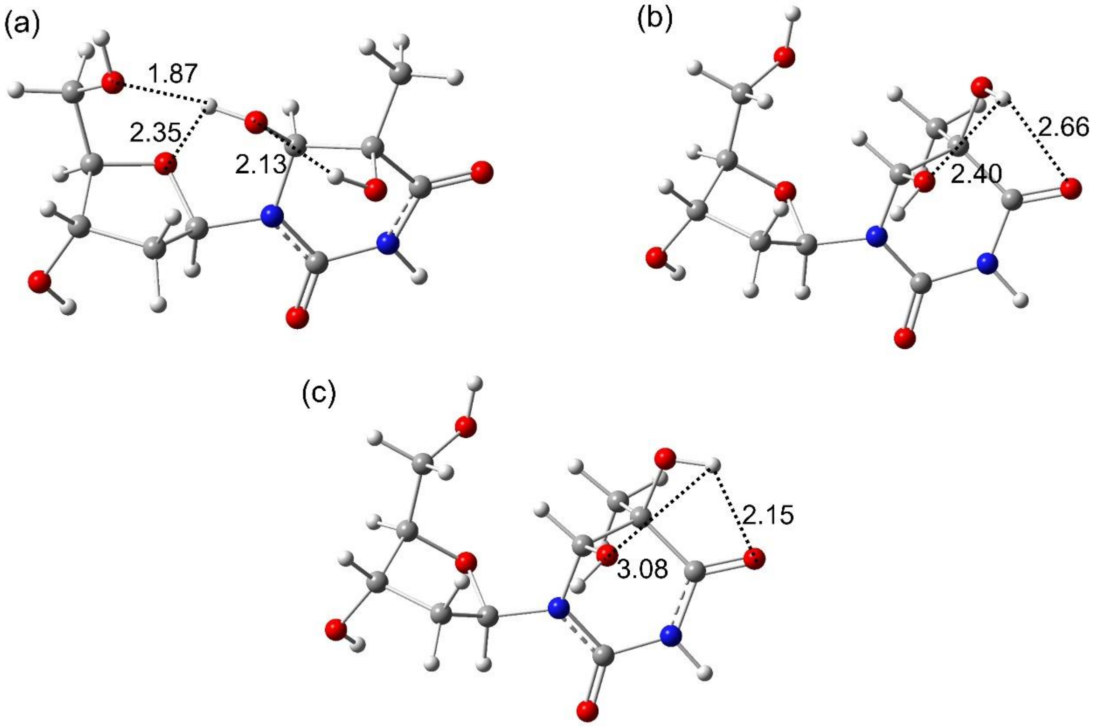

Fig. S5 Optimized structures of **5R6S-Tg. (a)** CH_3 in equatorial conformation; **(b)** CH_3 in axial conformation forming a **Tg**:O5H_{O5}•••O6:**Tg** hydrogen bond; **(c)** CH_3 in axial conformation forming a **Tg**:O5H_{O5}•••O4:**Tg** hydrogen bond. Key structural parameters are provided. Hydrogen bond lengh in Å.

Table S1. Single-point energies (kcal mol^{-1}) of **5R,6S-Tg** calculated at different level. Labels a, b and c refer to the structures in Figure S5.

	M062X/6-31+G(d,p)	MP2/6-311G(d,p)
a	0.00	0.00
b	3.56	3.50
c	3.51	3.49

Table S2 Interaction energy decomposition (kcal mol^{-1}) of T and **5R,6S-Tg** with its adjacent bases G5, G7 and A19 (*cf.* Figure 4).

Interaction energy	T			5R,6S-Tg		
	G5	G7	A19	G5	G7	A19
Elec	0.7±1.2	-0.7±1.2	-10.9±1.9	-1.5±1.6	2.6±1.7	-10.9±2.1
Vdw	-6.9±0.9	-5.3±0.9	-0.3±1.5	-7.6±0.6	-5.1±0.6	-0.6±1.4
Total	-6.2±1.4	-6.0±1.6	-11.2±1.1[a]	-8.9±1.5	-2.5±1.5	-11.5±1.4[b]

[a,b] The corresponding interaction energy calculated at M06-2X/6-31+G(d,p) are -13.8 kcal mol^{-1} and -12.9 kcal mol^{-1}, respectively.

Table S3 Interaction energy decomposition of T and **5R,6S-Tg** with the adjacent G5, G7 and A19 (kcal mol^{-1}) for replicas 2 and 3.

Interaction energy	DNA-thy[a]			DNA-thy[b]			cis-DNA[a]			cis-DNA[b]		
	G5	G7	A19	G5	G7	A19	G5	G7	A19	G5	G7	A19
Elec	0.8±1.3	-1.0±1.0	-11.0±1.8	0.8±1.3	-1.1±1.1	-11.0±1.8	-1.7±1.6	2.5±1.8	-11.0±2.2	-2.0±2.0	2.6±1.7	-10.8±2.1
Vdw	-6.7±0.9	-5.4±0.8	-0.2±1.4	-6.8±1.0	-5.5±0.8	-0.3±1.3	-7.3±0.7	-5.1±0.6	-0.6±1.4	-7.0±0.9	-5.1±0.6	-0.7±1.4
Total	-5.9±1.4	-6.4±1.5	-11.2±1.1	-6.0±1.3	-6.6±1.6	-11.3±1.1	-9.0±1.5	-2.6±1.5	-11.6±1.4	-9.2±1.9	-2.5±1.5	-11.5±1.3

[a] Replica 2; [b] Replica 3.

Table S4 Interaction energy decomposition of **5R,6R-Tg** with the adjacent G5, G7 and A19 (kcal mol^{-1}). (*cf.* Figure 8).

Interaction energy	metastable structure			trans-DNA-1				trans-DNA-2			trans-DNA-3		
	G5	G7	A19	G5	G7	A19	C20	G5	G7	A19	G5	G7	A19
Elec	-1.4±1.8	0.7±1.9	-7.0±2.5	0.8±0.8	-0.7±1.3	-8.5±2.4	-12.6±3.0	-0.2±0.6	-1.9±2.0	-8.2±2.1	-0.5±1.3	2.3±1.5	-10.6±1.9
Vdw	-1.4±1.3	-4.4±0.9	-1.6±1.4	-3.7±0.5	-3.5±0.8	-1.3±1.3	-3.2±1.2	-2.1±0.5	-2.2±1.3	-4.6±1.3	-5.5±1.0	-5.1±0.6	-0.6±1.4
Total	-2.8±2.1	-3.7±1.7	-8.6±1.7	-2.9±0.9	-4.2±1.4	-9.8±1.7[a]	-15.8±2.6[a]	-2.3±0.6	-4.1±2.8	-12.8±1.7[b]	-6.0±1.5	-2.8±1.4	-11.2±1.1[c]

[a] the corresponding interaction energy of Tg with A19/C20 calculated at M06-2X/6-31+G(d,p) is -26.3 kcal mol^{-1}; [b,c] the corresponding interaction energy calculated at M06-2X/6-31+G(d,p) are -13.4 and -13.7 kcal mol^{-1}, respectively.

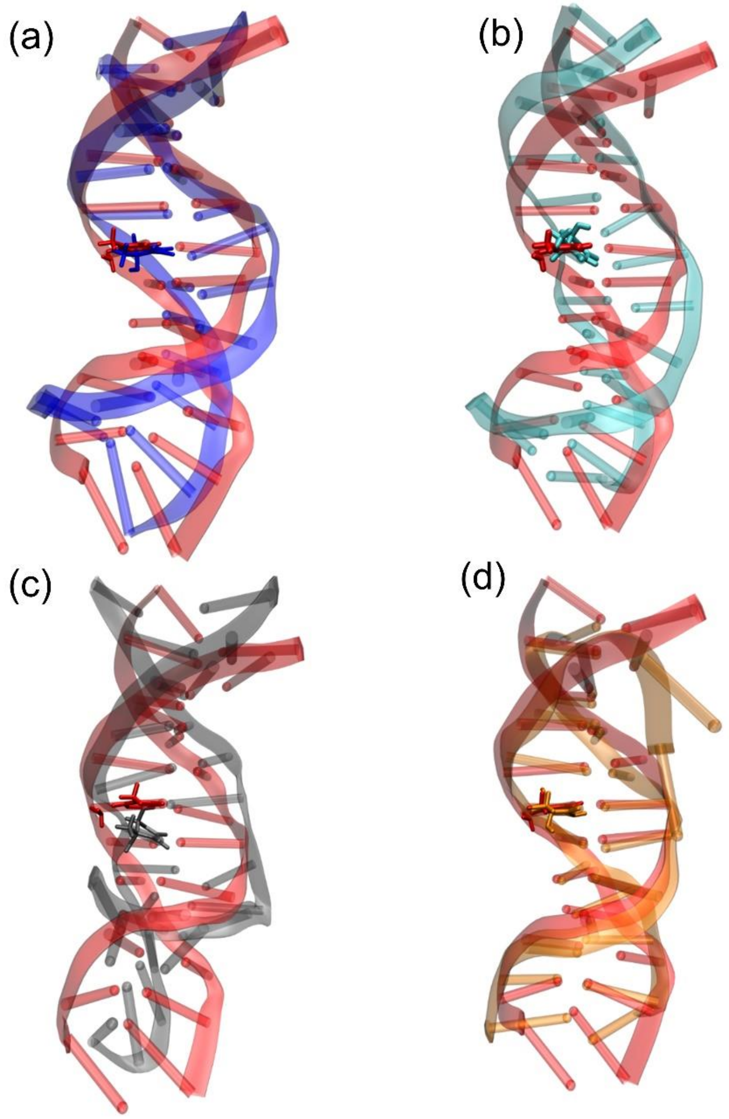

Fig. S6 Overlap of average structurse. **(a) DNA-cis** (in blue) to the intact **DNA-thy** (in red)**; (b) trans-DNA-1** (in cyan) to the intact **DNA-thy** (in red)**; (c) trans-DNA-2** (in silver) to the intact **DNA-thy** (in red)**; (d) trans-DNA-3** (in orange) to the intact **DNA-thy** (in red).

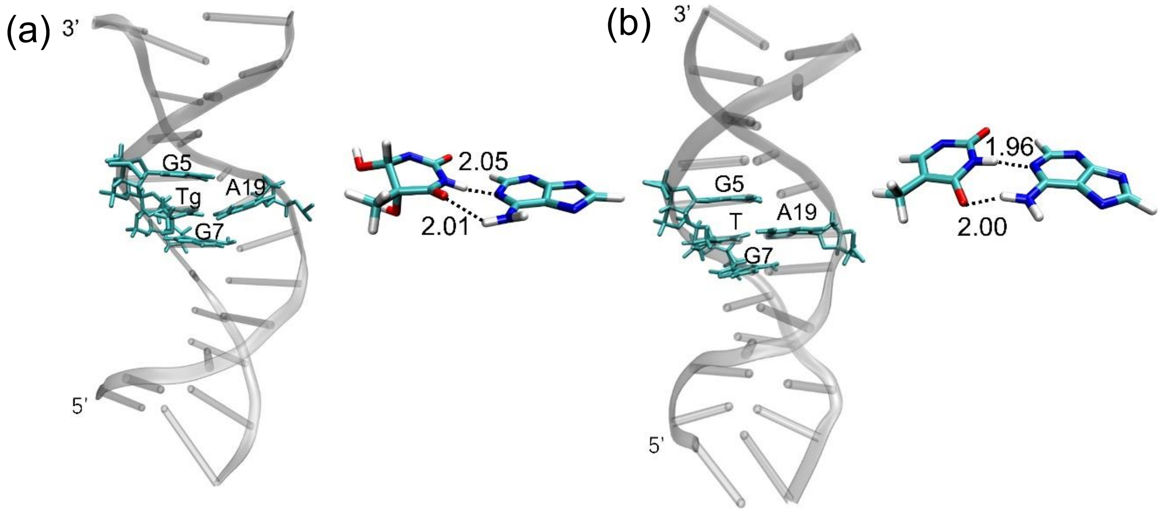

Fig. S7. The average structures of **(a)cis-DNA** and **(b)DNA-thy**. Left: placement of the structures in the DNA duplex; right: zooming in on the hydrogen bonded Tg-A19 base pairs.

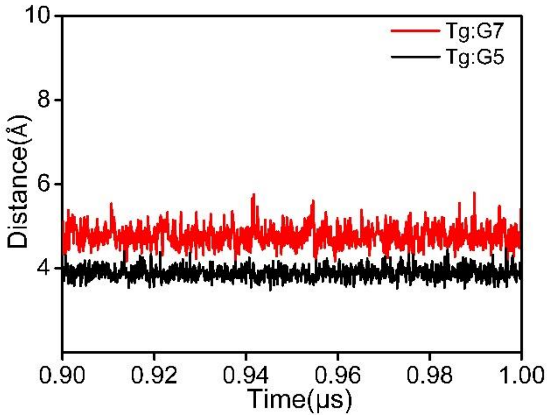

Fig. S8 The centroid distance of **cis-5R,6S-Tg** to G5 (3.89±0.15) and G7 (4.78±0.25) during the last 100 ns simulation of **cis-DNA**.

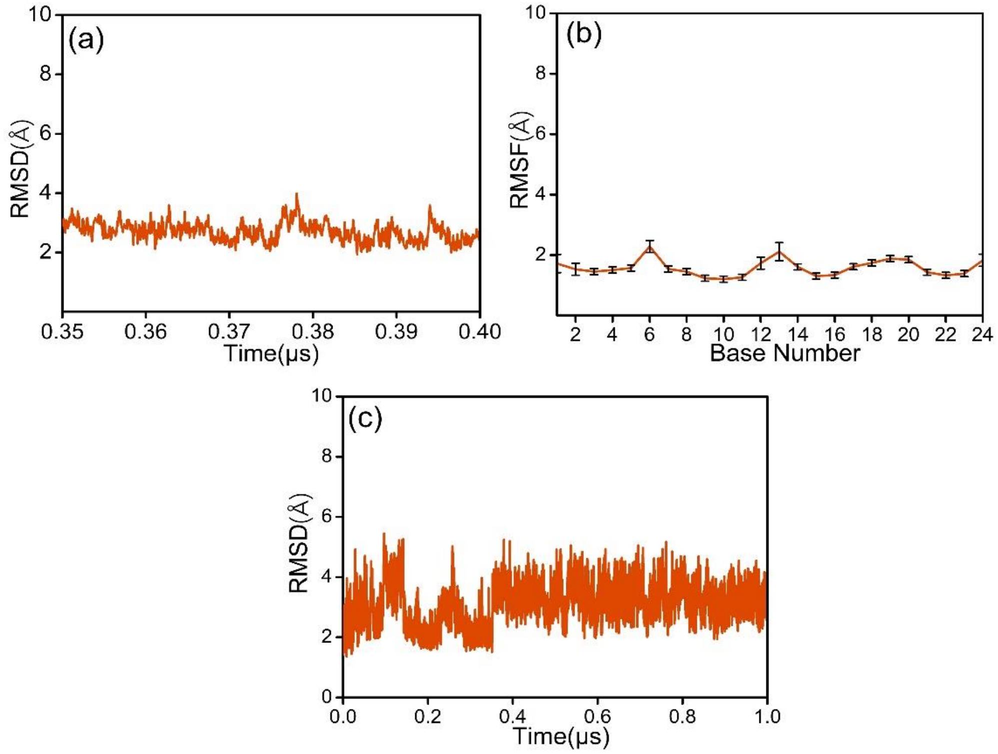

Fig. S9 (a) RMSD (2.90±0.30) and **(b)** RMSF of the **trans-DNA-1** bases during the 0.35-0.40 μs simulation; **(c)** RMSD (3.11±0.64) for the total 1 μs simulation of **trans-DNA-1**.

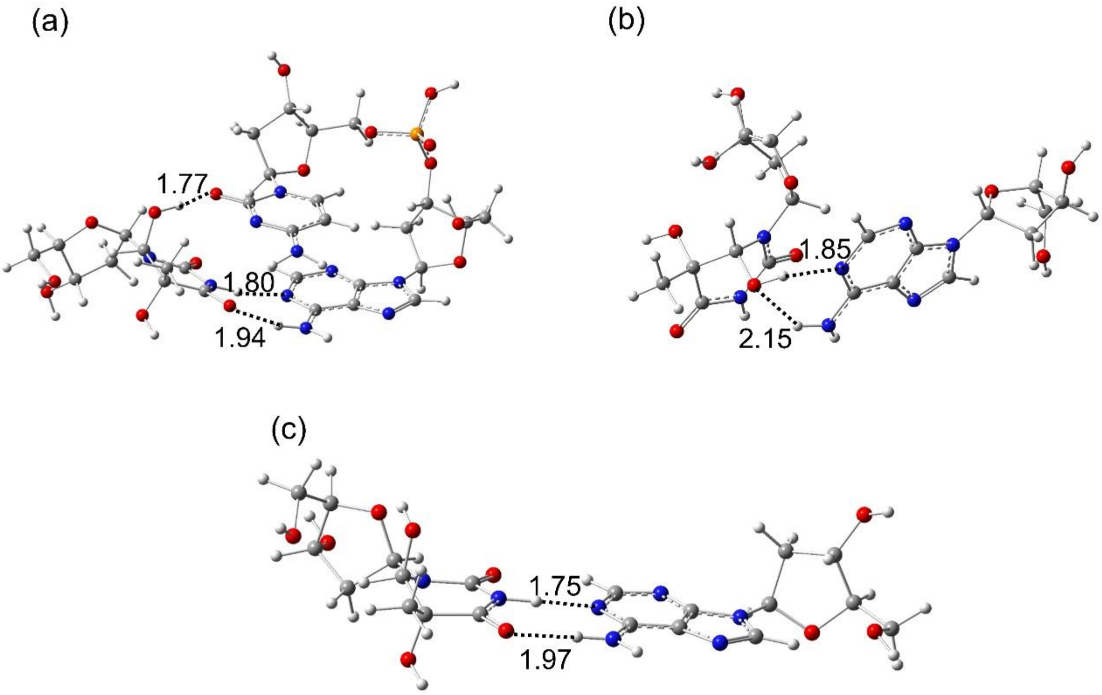

Fig. S10 (a) Optimized structures of **5R6R-Tg** with A19/C20 for **trans-DNA-1**; **(b)** Optimized structures of **5R6R-Tg** with A19 for **trans-DNA-2**; **(c)** Optimized structures of **5R6R-Tg** with A19 for **trans-DNA-3**. Hydrogen bond lenghs in Å.

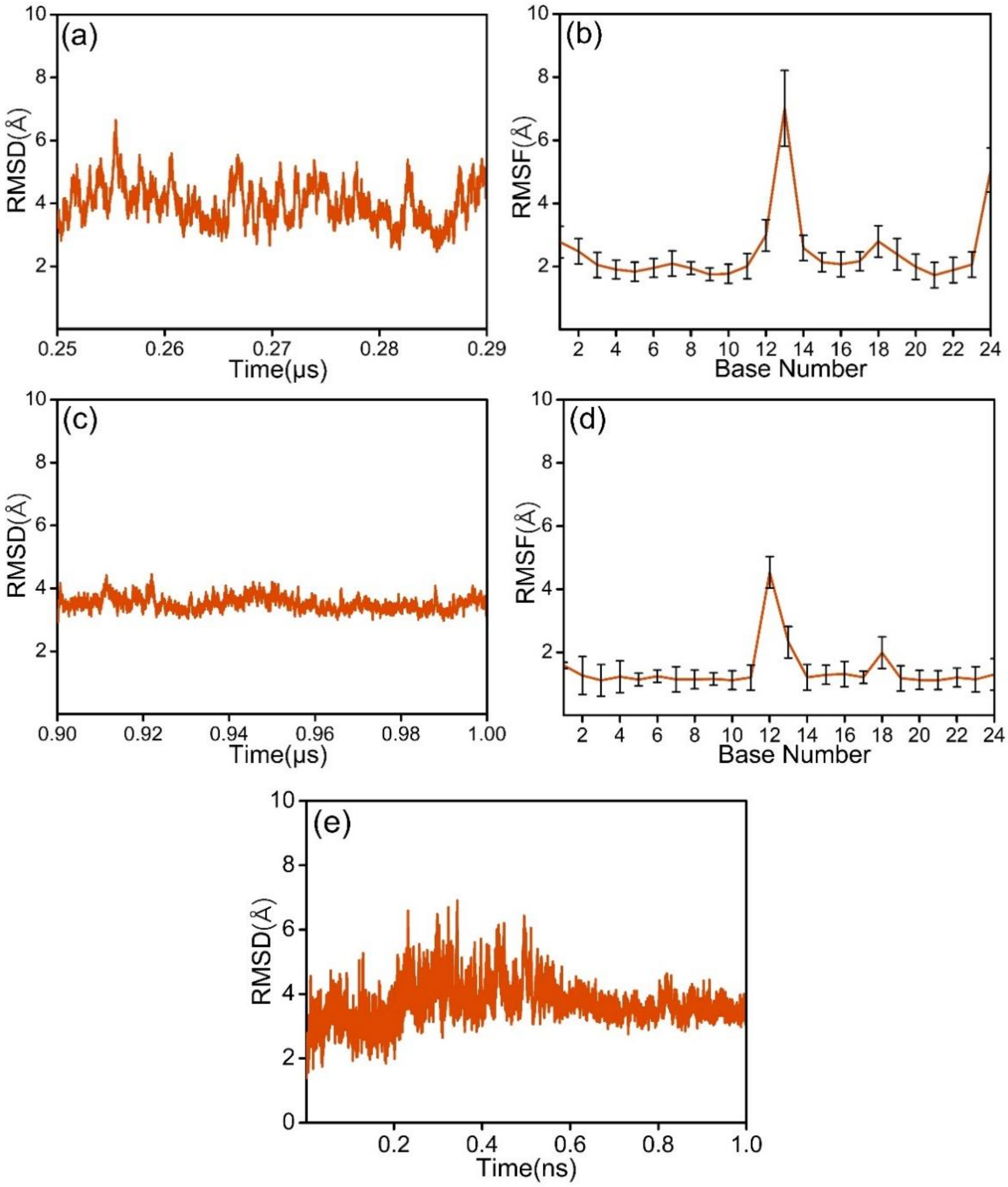

Fig. S11 (a) RMSD (4.00±0.62) and **(b)** RMSF for the metastable state (0.25-0.290 μs) of **trans-DNA-2**; **(c)** RMSD (3.51±0.21) and **(d)** RMSF of **trans-DNA-2** of the last 0.1 μs simulation; **(e)** The RMSD (3.70±0.64) of the total 1 μs simulation of **trans-DNA-2.**

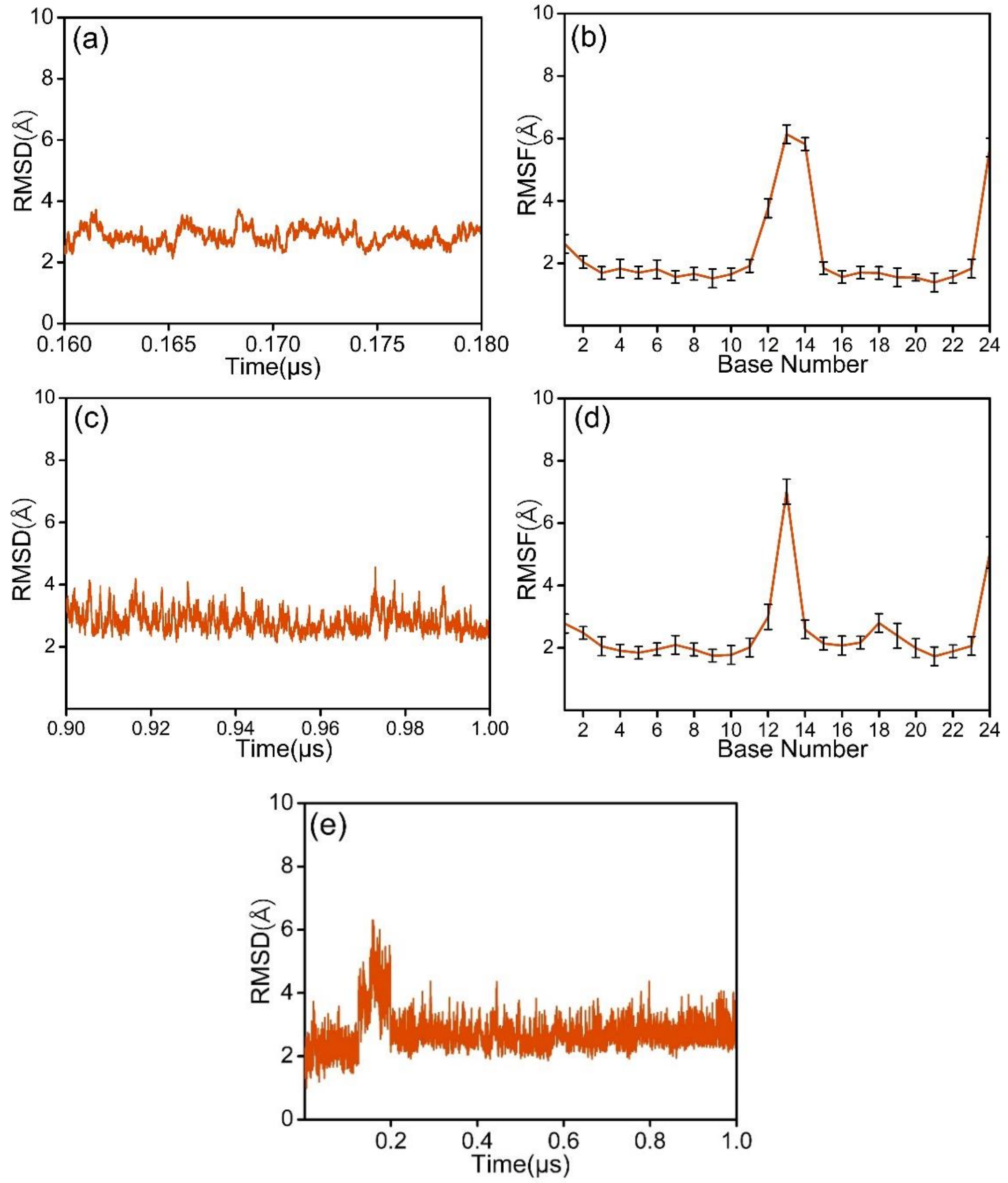

Fig. S12 (a) RMSD (2.86±0.28) and **(b)** RMSF for the metastable state (0.16-0.18 μs) of **trans-DNA-3**; **(c)** RMSD (2.83±0.33) and **(d)** RMSF of **trans-DNA-3** of the last 0.1 μs simulation; **(e)** The RMSD (2.73±0.56) of the total 1 μs simulation of **trans-DNA-3.**

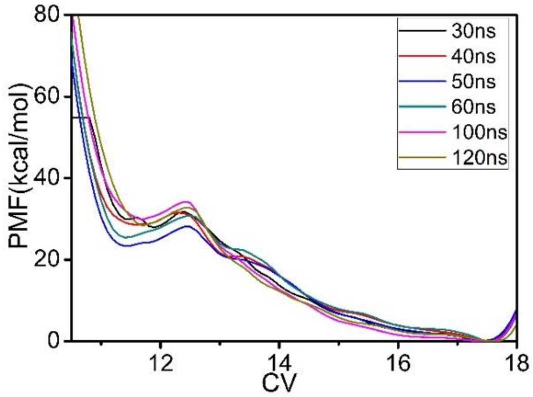

Fig. S13 Free energy profiles as a function of the CV between 5R6S-Tg and A19 for different flip times, to assess the convergence of the meta-eABF simulations.

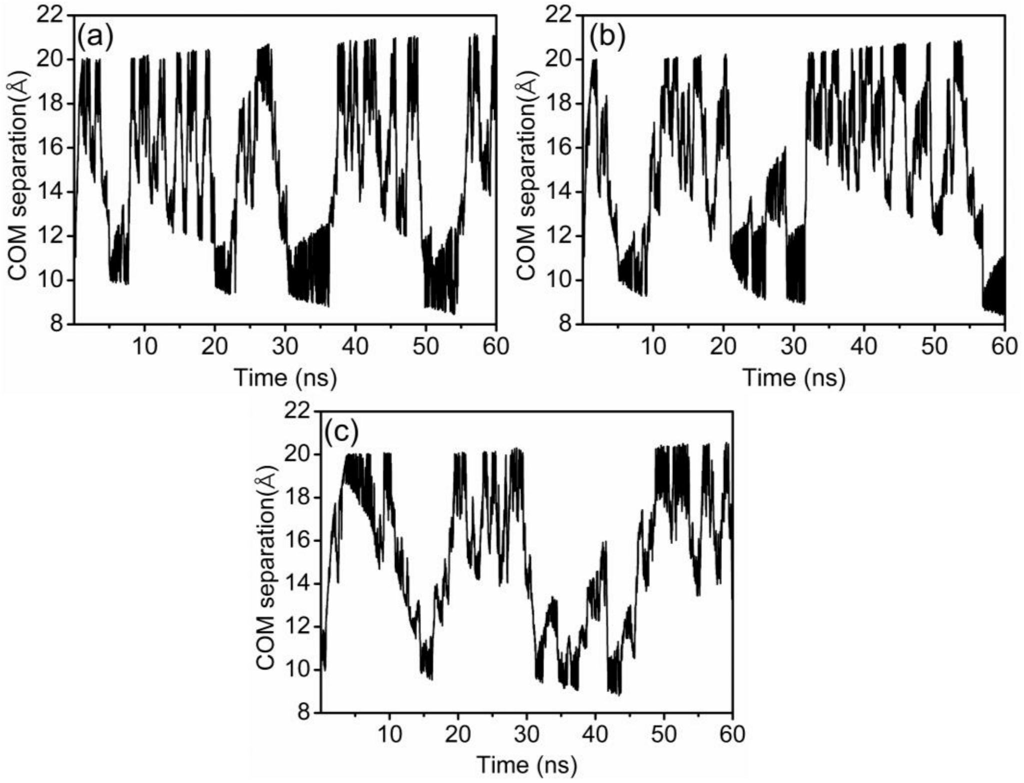

Fig. S14 Time evaluation plot of the examined CV of the meta-eABF simulations of the **(a) 5R6S-Tg** (**cis-DNA-1**); **(b)** native T (**DNA-Thy**) and **(c) 5R6R-Tg** (**trans-DNA-1**).

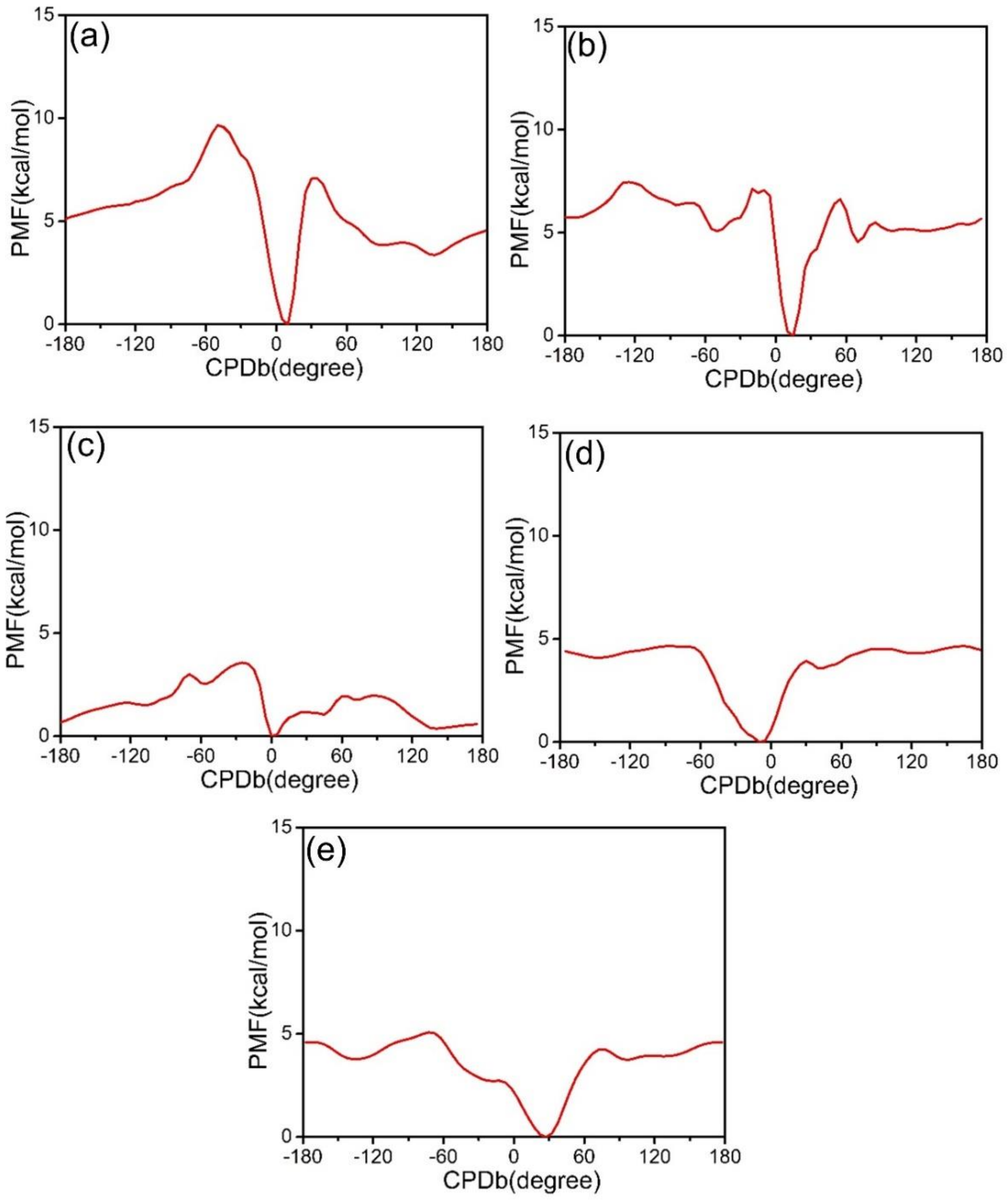

Fig. S15 PMF profiles of **T/Tg** flipping out of the duplex with pseudo-dihedral angle CPDb as the reaction coordinates. **(a)** 7.1 kcal mol^{-1} for **T** flipping in **DNA-thy** ; **(b)** 6.6 kcal mol^{-1} for **cis-5R,6S-Tg** in **cis-DNA**; **(c)** 1.9 kcal mol^{-1} for **trans-5R,6R-Tg** in **trans-DNA-1; (d)** 4.6 kcal mol^{-1} for **trans-5R,6R-Tg** in **trans-DNA-2**; **(e)** 5.1 kcal mol^{-1} for **trans-5R,6R-Tg** in **trans-DNA-3**. The barrier is based on the flipping of the base through major groove using 100 ns meta-ABF simulations. The CPDb dihedral angel are positive if Tg crosses into the major groove or negative if T/Tg crosses into the minor groove.

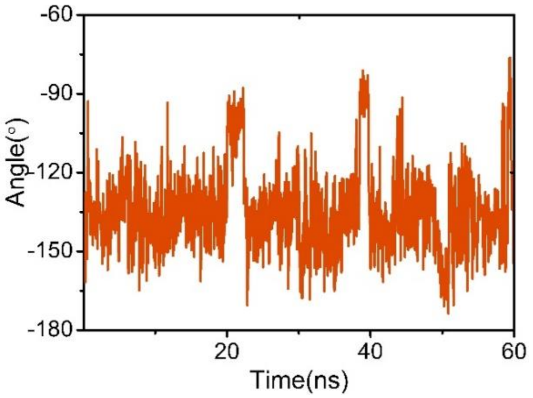

Fig. S16 Change in torsion angle C2-N3-C5-C5M during the 60ns meta-eABF simulations of **5R,6S-Tg** .

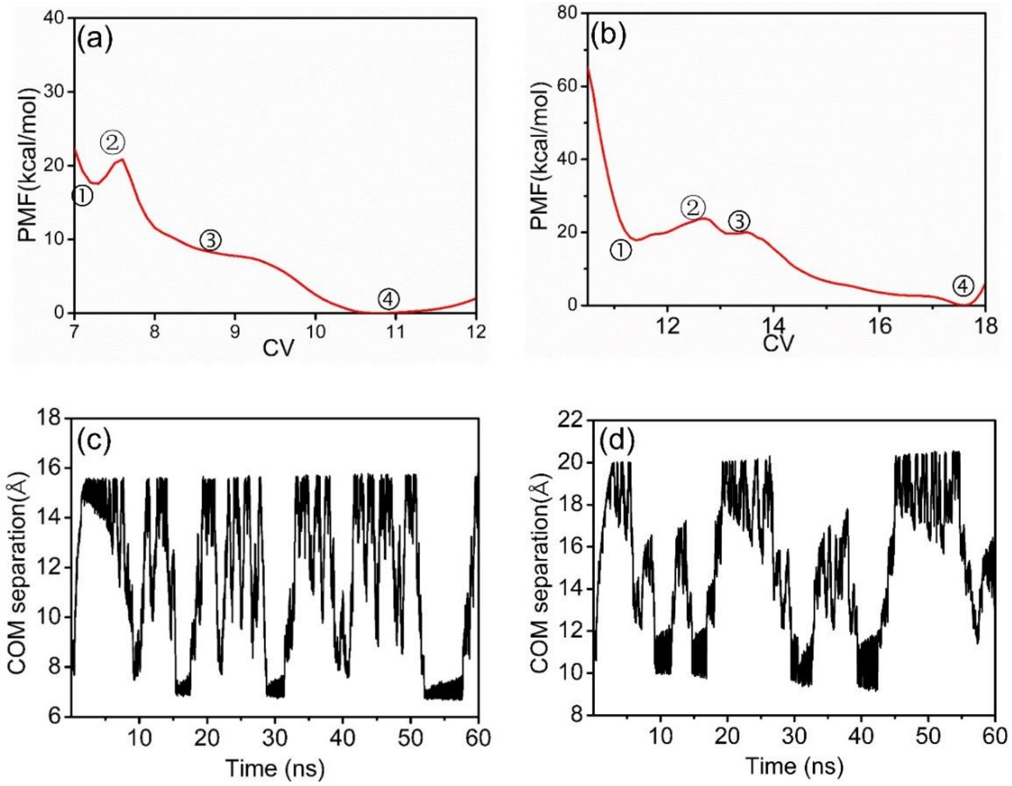

Fig. S17 PMF profiles of **5R6R-Tg** flipping out of the duplex. **(a) 4.1** kcal mol^{-1} for **trans-5R,6R-Tg** in **trans-DNA-2**; **(b) 5.2** kcal mol^{-1} for **trans-5R,6R-Tg** in **trans-DNA-3**, **(c), (d)** are the corresponding time evaluation plot of the examined CV distribution of the meta-eABF simulations.

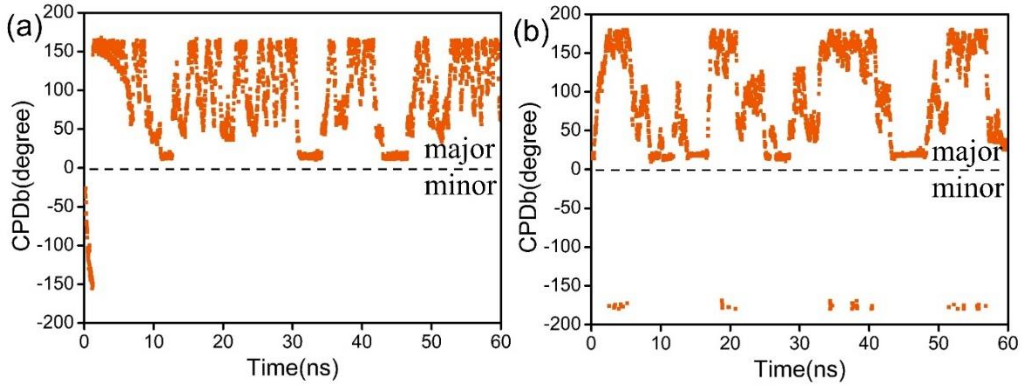

Fig. S18. The CPDb dihedral angle distribution of the Tg flipping out of the duplex during the simulation. **(a) trans-5R,6R-Tg** in **trans-DNA-2**; **(b) trans-5R,6R-Tg** in **trans-DNA-3.** The CPDb dihedral angle is positive if Tg crosses into the major groove and negative if T/Tg crosses into the minor groove.

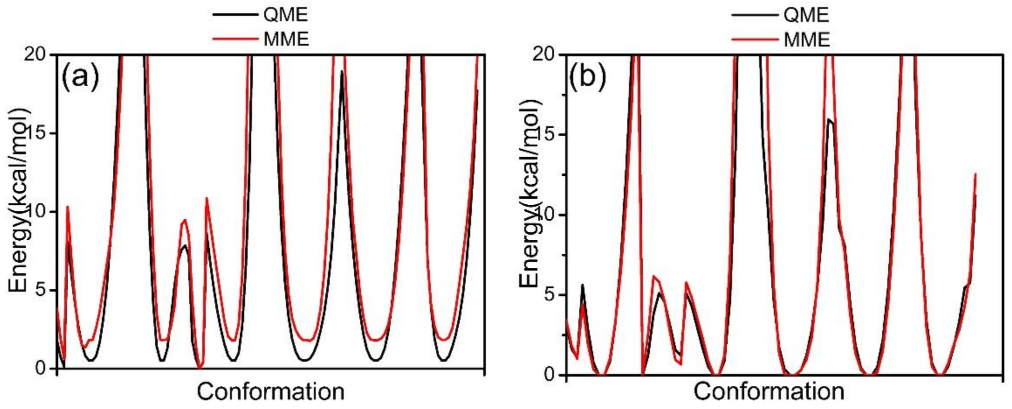

Fig. S19 Comparison of QM (black) and MM (red) potential energies of dihedral scans performed for **5R6S-Tg** (left) and **5R6R-Tg** (right) in order to optimize the dihedral parameters.

Table S5 Helical, backbone and groove parameters calculated with Curves+.

	Xdisp	Ydisp	Inclin	Tip	Ax-bend				
DNA-thy	0.76	0.89	11.5	2.1	1.6				
cis-DNA	-0.93	-0.65	12.8	-4.2	3.0				
trans-DNA-1	-2.00	-0.91	-5.7	2.8	2.9				
trans-DNA-2	2.47	0.79	-18.8	-14.5	7.5				
trans-DNA-3	0.83	-0.08	-1.9	7.2	4.8				
	Shear	Stretch	Stagger	Buckle	Prople	Opening			
DNA-thy	0.16	-0.16	0.03	3.1	-11.1	-0.2			
cis-DNA	-0.01	0.02	-0.03	-28.4	-9.6	5.3			
trans-DNA-1	0.3	0.65	1.34	-46.2	7.9	25.7			
trans-DNA-2	0.45	-2.57	4.31	-46.9	-5.1	4.4			
trans-DNA-3	0.28	-0.06	1.25	-18.9	-7.2	3.2			
	Shift	Slide	Rise	Tilt	Roll	Twist	H-rise	H-Twi	
DNA-thy	-0.37	2	3.74	0.4	-4.1	43	4	42.6	
cis-DNA	-0.07	0.27	3.27	0.3	7.7	29.5	3.21	30.2	
trans-DNA-1	0.34	-1.43	2.3	17.3	7.6	6.8	2.24	5.7	
trans-DNA-2	-1.58	-1.98	6.32	-0.8	-42.7	-13.6	4.16	-10.3	
trans-DNA-3	-1.01	2.12	3.6	3.9	-14.2	44.4	3.59	44.4	
	α	β	γ	δ	ε	ζ	χ	Pha	Amp
DNA-thy	-61.2	-175.7	44.3	142.1	-90.3	178.4	-93.1	155.8	41.5
cis-DNA	-83	173.3	63.3	86.5	-171	-81.4	-142.7	25.6	36.2
trans-DNA-1	-58.3	-172.2	43.6	80.5	-164	-54.6	-169.1	5.8	49.6
trans-DNA-2	-60.2	-178.8	45.6	86.4	-92.3	-158.9	-162.9	0.2	45.4
trans-DNA-3	-72.1	-149.3	47.3	138.3	-125.4	161	-81.1	171.3	41.4
	Min-W[a]	Min-D[b]	Maj-W[a]	Maj-D[b]					
DNA-thy	7.9	5	11.4	4.8					
cis-DNA	7.6	4.9	11.6	6.4					
trans-DNA-1	9.7	0.8	18.6	8					
trans-DNA-2	10.2	0.3	13.5	5.5					
trans-DNA-3	5.6	6.3	11.7	3.2					

[a] denote the width of the groove.

[b] denote the depth of the groove.
